# Endothelial destabilization by angiopoietin-2 via integrin β1 activation

**DOI:** 10.1038/ncomms6962

**Published:** 2015-01-30

**Authors:** Laura Hakanpaa, Tuomas Sipila, Veli-Matti Leppanen, Prson Gautam, Harri Nurmi, Guillaume Jacquemet, Lauri Eklund, Johanna Ivaska, Kari Alitalo, Pipsa Saharinen

**Affiliations:** 1 Wihuri Research Institute and Research Programs Unit, Translational Cancer Biology Program and Department of Virology, University of Helsinki, Biomedicum Helsinki, Haartmaninkatu 8, PO Box 63, Helsinki FI-00014, Finland; 2 Wihuri Research Institute and Research Programs Unit, Translational Cancer Biology, University of Helsinki, Biomedicum Helsinki, Haartmaninkatu 8, PO Box 63, Helsinki FI-00014, Finland; 3 Turku Centre for Biotechnology, University of Turku and VTT, Tykistökatu 6 A, Turku FI-20520, Finland; 4 Faculty of Biochemistry and Molecular Medicine, Oulu Center for Cell-Matrix Research, University of Oulu, PO Box 5400, Biocenter Oulu FI-90014, Finland

## Abstract

Angiopoietins regulate vascular homeostasis via the endothelial Tie receptor tyrosine kinases. Angiopoietin-1 (Ang1) supports endothelial stabilization via Tie2 activation. Angiopoietin-2 (Ang2) functions as a context-dependent Tie2 agonist/antagonist promoting pathological angiogenesis, vascular permeability and inflammation. Elucidating Ang2-dependent mechanisms of vascular destablization is critical for rational design of angiopoietin antagonists that have demonstrated therapeutic efficacy in cancer trials. Here, we report that Ang2, but not Ang1, activates β1-integrin, leading to endothelial destablization. Autocrine Ang2 signalling upon Tie2 silencing, or in Ang2 transgenic mice, promotes β1-integrin-positive elongated matrix adhesions and actin stress fibres, regulating vascular endothelial-cadherin-containing cell–cell junctions. The Tie2-silenced monolayer integrity is rescued by β1-integrin, phosphoinositide-3 kinase or Rho kinase inhibition, and by re-expression of a membrane-bound Tie2 ectodomain. Furthermore, Tie2 silencing increases, whereas Ang2 blocking inhibits transendothelial tumour cell migration *in vitro*. These results establish Ang2-mediated β1-integrin activation as a promoter of endothelial destablization, explaining the controversial vascular functions of Ang1 and Ang2.

## Abstract (web summary)

Angiopoietin-1 (Ang1) and angiopoietin-2 (Ang2) have opposing effects on vascular stability through their receptor Tie2, but there is evidence for Tie2-independent functions of Ang2. Here, Hakanpaa *et al.* show that Ang2 directly activates β1-integrin, leading to rearrangement of the actin cytoskeleton and decreased VE-cadherin in cell–cell junctions.

## Graphical abstract

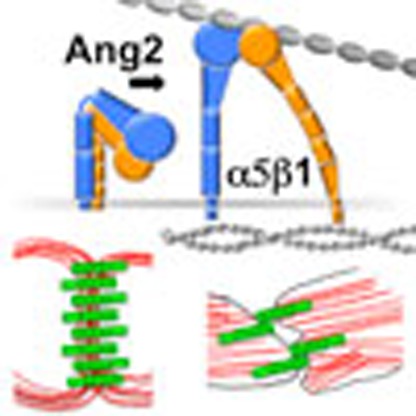

Angiopoietin growth factors, in addition to vascular endothelial growth factors (VEGFs), are critical regulators of vascular development, tissue homeostasis and pathological angiogenic responses^1^,^2^. Angiopoietin-1 (Ang1) mediates its vascular-stabilizing and anti-inflammatory effects by activating the endothelial Tie2 receptor^3^,^4^,^5^, while angiopoietin-2 (Ang2) is a weak Tie2 agonist whose activity is context dependent^6^,^7^. Ang2 levels, normally constrained during homeostasis, are elevated in endothelial cells during vessel remodelling, particularly in the tumour vasculature and in diseases associated with increased vascular permeability and endothelial dysfunction, such as sepsis and acute lung injury^8^,^9^. In addition to the increased Ang2/Ang1 ratio, decreased Tie2 levels have been reported in sepsis^10^,^11^. Such changes likely shift Ang1–Tie2 signalling in endothelial cell–cell junctions towards Ang2 signalling, thereby reducing Tie2 phosphorylation and priming the endothelium for inflammatory cytokine signals, permeability and vascular destablization^12^,^13^,^14^. Under these conditions, Ang2-blocking antibodies show beneficial vascular-stabilizing effects, demonstrating the critical role of Ang2 in processes leading to compromised vascular architecture^15^,^16^.

Ang2-blocking antibodies inhibit also tumour growth, tumour angiogenesis, as well as metastasis to lymph nodes and lungs^17^,^18^,^19^. In lung metastases, Ang2 inhibition improved, while transgenic endothelial Ang2 expression decreased capillary integrity, indicating that blocking Ang2 inhibits metastatic dissemination, in part, by enhancing the integrity of endothelial cell–cell junctions^19^. During vascular development, Ang2 inhibition blocked VE-cadherin phosphorylation at tyrosine residue 685 and the concomitant formation of button-like junctions in initial lymphatic vessels^20^. Transgenic expression of Ang2 in mouse embryos induced severe vascular abnormalities, including a discontinuous vascular network and collapsed endocardial lining of the heart^6^. These defects phenocopied those found in embryos lacking Tie2 or Ang1, indicating that Ang2 counteracted vascular stability promoted by Ang1–Tie2 signalling^3^,^6^,^21^. However, the vessel discontinuities caused by Ang2 overexpression were more severe than in Ang1 or Tie2 gene-targeted embryos, suggesting that Ang2 may have additional functions besides inhibiting the Ang1–Tie2 signals^6^.

Integrins, which regulate endothelial cell–cell and cell–matrix interactions, have been identified as alternative receptors for the angiopoietins in certain Tie2-negative non-endothelial cells^22^,^23^,^24^,^25^, and in the endothelial tip cells of angiogenic vessel sprouts, which express low levels of Tie2 (refs 26, 27). However, it remains unknown whether Ang1 and Ang2 differentially regulate integrin signalling, and whether integrins are involved in Ang2-induced endothelial destabilization. Here we report that Ang2, but not Ang1, directly activates β1-integrin. Autocrine Ang2 signalling in Tie2-silenced endothelial cells and in the Ang2 transgenic mice, which show reduced Tie2 localization in the endothelial cell–cell junctions, promotes changes in the cytoskeleton, matrix adhesion and cell junctions that lead to reduced cell–cell adhesion. These results suggest that Ang2, via β1-integrin activation, may predispose vessels to endothelial destablization.

## Results and Discussion

### Tie2 silencing reduces endothelial monolayer integrity

**Figure 1:**
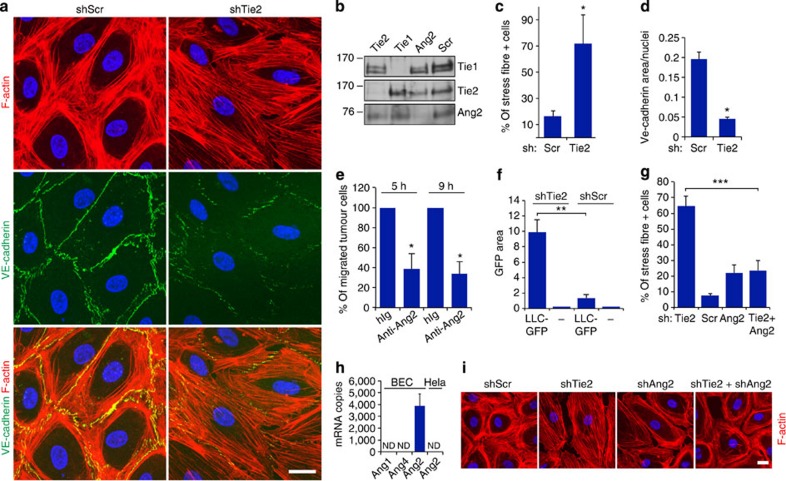
Ang2 reduces endothelial monolayer integrity in Tie2-dependent and -independent manners. (**a**) BECs were transduced with scramble (Scr) or Tie2 shRNA lentiviruses, fixed and stained for filamentous actin (F-actin) and VE-cadherin. (**b**) BECs were transduced with Scr, Tie2, Tie1 or Ang2 shRNA lentiviruses, and the cell lysates were analysed by western blotting using the indicated antibodies. (**c**) Quantification of the percentage of cells displaying actin stress fibres (% of stress fibre+ cells) (3 microscopic images/experiment, analysis of 60 cells/lentiviral transduction, *n*=3 independent experiments, *P*=0.04, Dunnet’s test). (**d**) Quantification of VE-cadherin area/nuclei in Scr or Tie2-silenced BECs (5 images/experiment, *n*=3 independent experiments, *P*=0.01, Dunnet’s test). (**e**) BECs seeded on fibronectin-coated Transwell inserts were treated with anti-Ang2 antibody or control hIg, and the GFP-expressing LNM-35 cancer cells transmigrated in 5 or 9 h were counted (percentage of transmigrated anti-Ang2 versus control antibody-treated cells, *n*=3 independent experiments, each performed in triplicate, *P*=0.05 (5 h), *P*=0.01 (9 h), Student’s *T*-test). (**f**) LLC-GFP cancer cells transmigrated during 9 h through a BEC monolayer transduced with Scr or Tie2 shRNA lentiviruses were quantified as the GFP-positive area (*n*=3 independent experiments, each in duplicate or triplicate, *P*=0.007, Student’s *T*-test). (**g**) BECs were transduced with Scr, Tie2, Ang2 or Tie2+Ang2 shRNA lentiviruses, fixed and stained for F-actin. Quantification of the percentage of cells displaying actin stress fibres (5–7 microscopic images/experiment, 500 cells/transduction analysed, *n*=4 independent experiments, *P*=0.001, Dunnet’s test). (**h**) Q-RT–PCR analysis of Ang1, Ang2 and Ang4 messenger RNA (mRNA) expression in BECs and HeLa cells. Shown is mRNA copy number in 10 ng total RNA. ND=mRNA not detected. (**i**) Representative images of Scr, Tie2, Ang2 and Tie2+Ang2-silenced BECs. Mean and s.d. *P<0.05, **P<0.01, ***P<0.005. Scale bars, 20 μm. Nuclear 4′,6-diamidino-2-phenylindole stain. Confocal microscopic images.

To investigate the function of endothelial cell-secreted Ang2 on endothelial integrity, we silenced Ang2, Tie2 or the related Tie1 receptor in human dermal blood microvascular endothelial cells (BECs), which secrete endogenous Ang2 stored in Weibel–Palade bodies^28^. In BECs transduced with scrambled (Scr) control short hairpin RNA (shRNA) lentivirus (Fig. 1a) and in non-transduced BECs (Supplementary Fig. 1b), actin formed a peripheral cortical structure, which stabilizes endothelial cell–cell junctions and monolayer integrity^29^. Tie2 silencing (shTie2) induced an elongated cell morphology, loss of the cortical actin rim and led to the formation of prominent actin stress fibres extending across the cell body (Fig. 1a–c; Supplementary Fig. 1). In contrast, Ang2 or Tie1 silencing had minimal effects on the cortical actin cytoskeleton (Fig. 1g,i; Supplementary Fig. 1a,c). In line with the known role of actin stress fibres in generating a centripetal tension that weakens endothelial cell–cell junctions^30^, the adherens junction proteins VE-cadherin and β-catenin were reduced in the cell–cell contacts of Tie2-silenced BECs and human pulmonary microvascular endothelial cells (Fig. 1a,d; Supplementary Fig. 2a,b). Importantly, Tie2 silencing reduced cell surface VE-cadherin more than total VE-cadherin (Supplementary Fig. 2c). However, the tight junction protein ZO-1 was not affected by Tie2 silencing (Supplementary Fig. 2d).

We recently reported that Ang2-blocking antibodies improve the integrity of endothelial cell–cell junctions in the lungs of tumour-bearing mice, thereby contributing to reduced pulmonary metastasis in mouse models^19^. To test whether Ang2 was directly involved in the regulation of transendothelial tumour cell migration, we employed BEC monolayers grown on Transwell inserts. Ang2-blocking antibodies, targeting the Ang2–Tie2 interaction, inhibited the migration of fluorescently labelled human lung carcinoma (LNM-35) cells across the endothelial cell monolayer, but did not affect Ang2 secretion by BECs, or LNM-35 cell migration in the absence of endothelial cells (Fig. 1e; Supplementary Fig. 2e,f). In contrast, the Tie2-silenced endothelium was significantly more permissive for transmigration of green fluorescent protein (GFP)-tagged murine Lewis lung carcinoma (LLC) cells than the control-transduced endothelium (Fig. 1f), indicating a compromised endothelial barrier in the absence of Tie2, in line with the observed decrease in adherens junction proteins in the Tie2-silenced cells (Fig. 1a,d; Supplementary Fig. 2a,b).

### Ang2 regulates endothelial integrity independently of Tie2

Ang2 is the only Tie2 ligand expressed to significant levels in BECs according to results of messenger RNA profiling and quantitative reverse transcription (Q-RT)–PCR (Ang1/Tie2 ratio 0.01; Ang2/Tie2 ratio 6.8 (ref. 31); Fig. 1h). We envisioned that Ang2 signalling may be deregulated upon silencing of its receptor Tie2, leading to altered cellular architecture and increased tumour cell migration; we therefore simultaneously silenced Tie2 and Ang2 in the BECs. The alterations in actin architecture (that is, loss of cortical actin and the formation of prominent actin stress fibres) following Tie2 silencing were rescued upon Ang2+Tie2 double silencing (Fig. 1g,i; Supplementary Fig. 2g). These data indicated that endothelial Ang2 stimulates stress fibre formation via a Tie2-independent mechanism.

### Ang2 regulates β1-integrin activation in Tie2-silenced cells

**Figure 2:**
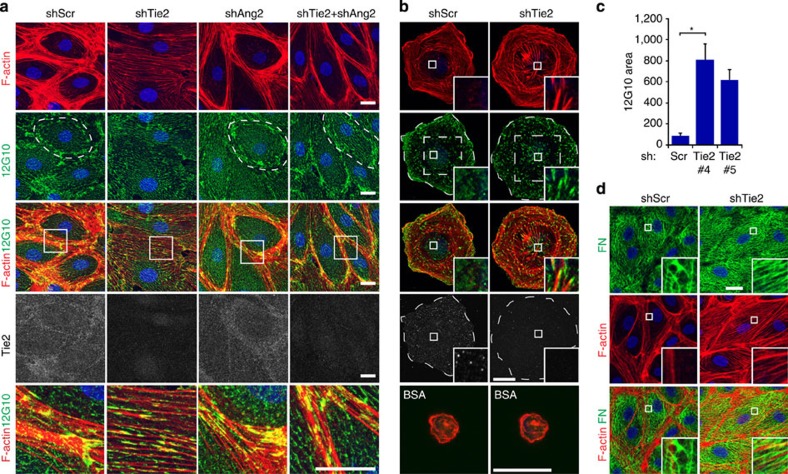
Ang2 promotes β1-integrin activation and interferes with fibronectin fibrillogenesis in Tie2-silenced endothelial cells. (**a**) BECs were transduced with scramble (Scr), Tie2, Ang2 or Tie2+Ang2 shRNA lentiviruses, fixed and stained for filamentous actin (F-actin), active β1-integrin (12G10) and Tie2. Note the presence of central, active β1-integrin-positive elongated matrix adhesions in Tie2-silenced BECs, and the peripheral localization of active β1-integrin, overlapping with cortical actin in Scr, Ang2 and Tie2+Ang2 double-silenced cells (dashed circle). Magnification of the boxed area is shown in the lowest row. (**b**) BECs transduced with Scr or Tie2 shRNA lentiviruses were allowed to adhere for 30 min on fibronectin or BSA, fixed and stained for F-actin, active β1-integrin and Tie2. Note the presence of active β1-integrin-positive matrix adhesions in the central half of the total cell area (boxed area, dashed line) in Tie2-silenced, but not in Scr-transduced, BECs. (**c**) Quantification of β1-integrin-positive matrix adhesion sites normalized to the central 50% of cell area used for quantification (*n*=3 independent experiments for Scr and Tie2#4, 45 cells/transduction analysed, *P*=0.01; *n*=2 for Tie2#5, 30 cells analysed, 10 microscopic fields/experiment analysed, *P*=0.07). Mean and s.d. *P<0.05. Student’s *T*-test. (**d**) BECs on vitronectin were transduced with Scr or Tie2 shRNA lentiviruses in growth media containing fibronectin-depleted serum. The cells were stained for F-actin and fibronectin. Nuclear Hoechst stain. Projections of confocal z-stacks. Scale bars, 20 μm.

Integrins, which are known to regulate endothelial cell–cell adhesion by the reorganization of the cell cytoskeleton^32^, have been previously reported to interact with angiopoietin–Tie2 receptor complexes^33^,^34^ and to function as alternative receptors for angiopoietins^23^,^24^,^25^,^27^. As BECs express high levels of both β1- and α5-integrins, which function as an α5β1 heterodimer to efficiently bind RGD-containing fibronectin, we first investigated whether β1-integrin activity was altered in the Tie2-silenced cells by using β1-integrin activation-state-specific antibodies (12G10 and 9EG7). In BECs growing on fibronectin, active β1-integrin was localized in focal adhesions in the cell periphery close to the cell–cell junctions (Fig. 2a; Supplementary Fig. 3a). However, in the Tie2-silenced BECs, active β1-integrin was detected centrally and across the cell body in prominent, elongated adhesion structures, resembling fibrillar adhesions, indicative of increased cell retraction and altered substrate adhesion sites (Fig. 2a; Supplementary Fig. 3a). Furthermore, during cell spreading on fibronectin, we noted markedly enhanced generation of centrally located, active β1-integrin-containing adhesions in the Tie2-silenced cells, when compared with Scr-transduced cells (Fig. 2b,c; Supplementary Fig. 4). When both Tie2 and Ang2 were silenced in BECs the formation of the β1-integrin-positive elongated adhesions was inhibited, indicating that Ang2 was required for the increased β1-integrin activation in the cell centre (Fig. 2a).

Integrin α5β1 is involved in extracellular matrix deposition by endothelial cells^35^. Rho kinase-mediated stress fibre formation and traction forces promote the translocation of fibronectin-bound α5β1 integrins towards the cell body, enabling the unfolding of fibronectin cryptic sites and fibrillogenesis^36^. We therefore tested whether fibronectin remodelling was altered in the Tie2-silenced cells. Control cells, cultured on vitronectin in fibronectin-depleted growth medium, generated a dense network of fibrillar fibronectin, whereas the Tie2-silenced cells produced straight and parallel short fibronectin fibres aligned along the direction of the actin stress fibres (Fig. 2d), suggesting that increased β1-integrin activity altered fibronectin fibrillogenesis in Tie2-silenced BECs.

### Integrin-mediated regulation of actin cytoskeleton

**Figure 3:**
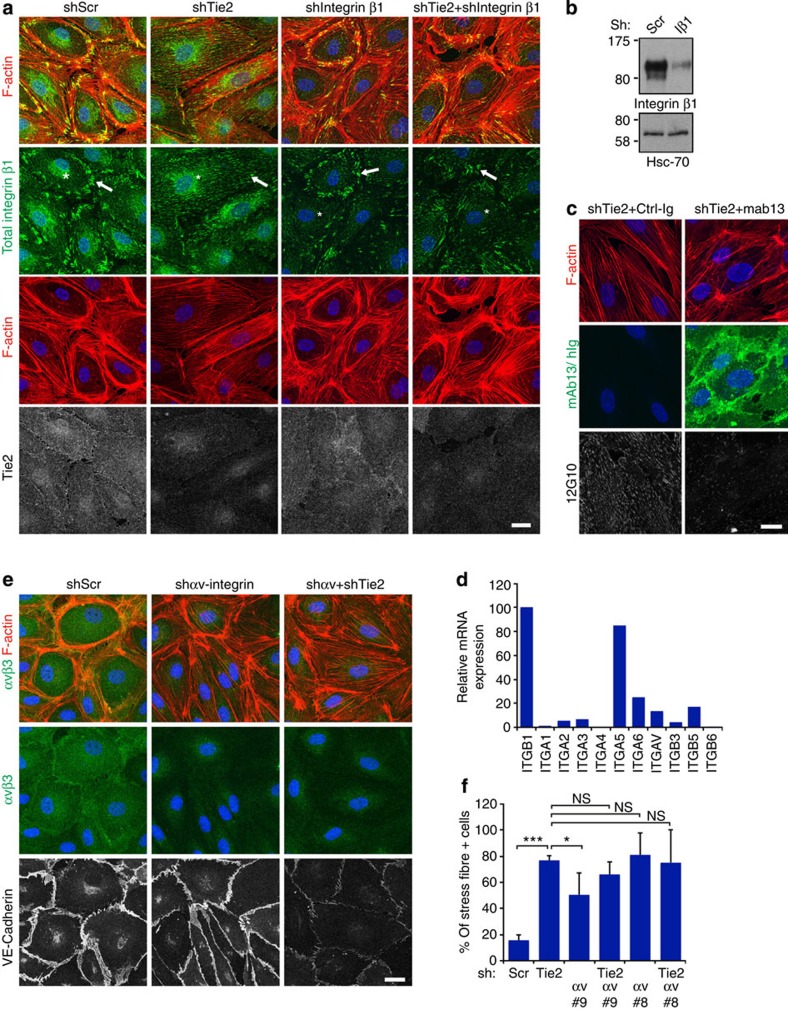
Silencing of β1-, but not αv-, integrin rescues cortical actin cytoskeleton in Tie2-silenced BECs. (**a**) BECs were transduced with scramble (Scr), Tie2, β1-integrin or Tie2+β1-integrin shRNA lentiviruses, fixed and stained for filamentous actin (F-actin), total β1-integrin and Tie2. β1-integrin-positive adhesions (arrows) are reduced, but not completely abolished in β1-integrin-silenced cells. Diffusely localized and perinuclear β1-integrin (asterisk) is not detected in β1-integrin-silenced cells. (**b**) Western blot of Scr and β1-integrin-silenced BEC lysates using the indicated antibodies. (**c**) BECs were transduced with Scr and Tie2 shRNA lentiviruses for 48 h, treated with control or β1-integrin-blocking antibodies (mab13) (10 μg ml^−1^) during 32–48 h after transduction and stained for F-actin and active β1-integrin. Mab13 was detected with anti-rat secondary antibodies. (**d**) Integrin expression in BECs using Q-RT–PCR, relative to β1-integrin expression (set as 100). (**e**) BECs were transduced with Scr, two different αv-integrin shRNA lentiviruses alone and in combination with Tie2 shRNA, fixed and stained for F-actin, αvβ3-integrin and VE-cadherin. (**f**) Quantification of the percentage of cells displaying actin stress fibres (% of stress fibre+ cells) in **e** (number of cells analysed/lentiviral transduction: 307/Scr; 229/Tie2; 242/αv-integrin#9; 315/αv-integrin#9+Tie2; 278/αv-integrin#8; 249/αv-integrin#8+Tie2, *n*=2). Mean and s.d. *P<0.05, ***P<0.005. NS=not significant, Dunnet’s test. Scale bars, 20 μm. Nuclear 4′,6-diamidino-2-phenylindole stain. Projections of confocal z-stacks.

We next investigated whether β1-integrin was required for the change in the cellular actin architecture observed in the Tie2-silenced BECs. β1-integrin silencing did not affect the cortical actin cytoskeleton of control cells, likely due incomplete β1-integrin silencing in these cells. However, β1-integrin silencing was effective in blocking the formation of the actin stress fibres in the Tie2-silenced BECs (Fig. 3a,b). Treatment with β1-integrin-blocking antibodies (mAb13, 4 μg ml^−1^), to more efficiently inhibit β1-integrin function in BECs, disrupted both actin stress fibres and the cortical actin rim of Tie2-silenced and control cells, respectively (Fig. 3c; Supplementary Fig. 3b). These results suggest that β1-integrin in the cell periphery maintains the endothelial cortical actin cytoskeleton, and that Ang2 promotes cytoskeletal changes via β1-integrin deposition in the central elongated adhesions, which support actin stress fibre formation.

To investigate the signalling mechanisms that contribute to the Ang2-β1-integrin-induced cytoskeletal rearrangement in endothelial cells, we analysed cell lysates using phosphoproteomic profiling (Supplementary Fig. 5a). Silencing of Ang2 had no significant effect on the phosphorylation of intracellular signalling proteins when compared with control shRNA-transduced cells. However, the Tie2-silenced cells, but not cells co-silenced for Tie2 and Ang2 or Tie2 and β1-integrin, showed prominent phosphorylation of Akt and Erk (Supplementary Fig. 5a,b). Akt is activated by PI3K and indeed, treatment with the PI3K inhibitor LY294002 prevented the stress fibre formation, rescuing the cortical actin rim, but not VE-cadherin-positive cell junctions, in the Tie2-silenced cells (Supplementary Fig. 5c). Similarly, inhibition of Rho kinase activity reduced stress fibres and promoted the formation of cortical actin structures in the Tie2-silenced cells (see Supplementary Fig. 5d,e).

In addition to β1-integrin, BECs express β3 and β5-integrins, which together with the αv-integrin, form RGD-binding heterodimers (Fig. 3d). In contrast to β1-integrin, the αvβ3 heterodimer was more evenly distributed in BECs and enriched in the cell–cell contacts in control, and to a lesser extent, in Tie2-silenced BECs (Supplementary Fig. 3c). Integrin αvβ5 was localized around the cell nucleus in confluent BECs, in line with previous results^37^, but was associated with cell adhesions at the ends of actin stress fibres in few Tie2-silenced cells (Supplementary Fig. 3c). Silencing of αv, β3 or β5-integrins did not rescue the cortical actin structure in the Tie2-silenced cells (Fig. 3e,f; Supplementary Fig. 3d–f). Furthermore, αv- and β3-integrin silencing resulted in disorganized cortical actin (Fig. 3e,f; Supplementary Fig. 3e), in line with previous reports implicating αvβ3 in the stabilization of the cortical actin cytoskeleton in endothelial cells^38^,^39^. Taken together, these data demonstrate that the cytoskeletal and junctional alterations induced by Tie2 silencing in BECs are not dependent on αv-integrins, and instead rely specifically on β1-integrin function.

### Ang2, but not Ang1, activates β1-integrin

**Figure 4:**
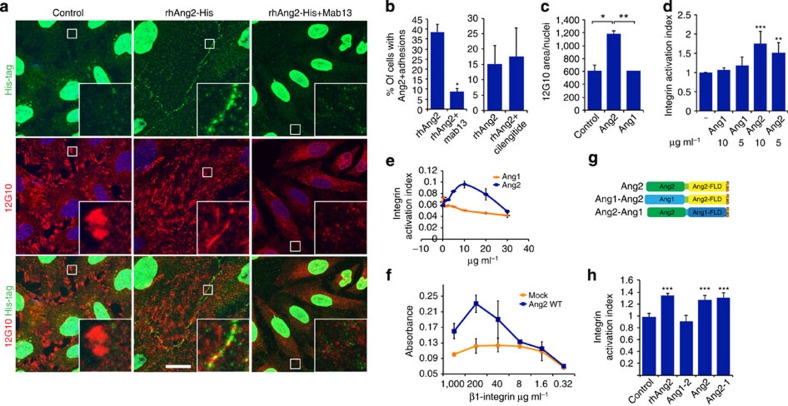
The N-terminal domain of Ang2, but not of Ang1, activates β1-integrin. (**a**–**c**) HeLa cells seeded on fibronectin were stimulated with 4 μg ml^−1^ rhAng2 (**a**,**b**) or rhAng1 (**c**) for 30 min, or pretreated with β1-integrin-blocking antibody (4 μg ml^−1^ mab13, **a**,**b**) or cilengitide (10 μM, **b**) for 5 min, and then further stimulated for 30 min with rhAng2. The cells were stained for active β1-integrin (12G10) and for His-tag (**a**,**c**) or Ang2 (**b**). (**b**) Ang2-positive matrix adhesions were analysed from 7 (mAb13) or 10 (cilengitide) microscopic images/experiment (total of 500 cells/mAb13 treatment and 320 cells/cilengitide treatment, *n*=3 for mAb13, for cilengitide a representative experiment is shown, repeated three times) *P*=0.03 for mAb13 and *P*=0.496 for cilengitide, Student’s *T*-test. (**c**) Active β1-integrin was quantified from 7 microscopic images/experiment, total of 350 cells/treatment analysed, *n*=3, *P*=0.04 for control versus Ang2, *P*=0.01 for Ang2 versus Ang1, *P*=0.99 for control versus Ang1, Dunnet’s test. (**d**) CHO cells were incubated with fluorescently labelled fibronectin fragment (FN7–10) and with various concentrations of rhAng2 or rhAng1, as indicated. FN7–10 binding to CHO cells was quantified using fluorescence-activated cell sorting, and normalized to total α5β1 levels, as explained in the methods. *P*=0.0004 for 10 μg ml^−1^ (150 nM) (*n*=3) and *P*=0.007 for 5 μg ml^−1^ (75 nM) (*n*=3) concentration of rhAng2 versus FN7–10 only, Dunnet’s test. (**e**) CHO cells were treated with the indicated concentrations of rhAng1 or rhAng2, and integrin activation measured as in **d**. (**f**) Binding of Ang2–Flag to biotinylated β1-integrin ectodomain was measured in triplicate using ELISA, a representative experiment of two independent experiments is shown. (**g**,**h**) CHO cells transduced with a control plasmid, or plasmids encoding for flag-tagged Ang2, Ang1–Ang2 (Ang1–2) or Ang2–Ang1 (Ang2–1) chimeric proteins (schematic structures are shown in **g**) were incubated with FN7–10 and, where indicated, with 10 μg ml^−1^ rhAng2. FN7–10 binding to CHO cells was quantified as in **d**. *P*=0.004 for rhAng2, *P*=0.002 for Ang2–Flag, *P*=0.001 for Ang2–1–Flag versus FN only, (*n*=3, Dunnet’s test). Mean and s.d. *P<0.05, **P<0.01, ***P<0.005. Nuclear Hoechst stain. Projections of confocal z-stacks. Scale bar, 20 μm.

Our results suggested that when Tie2 levels are downregulated in endothelial cells, the subsequent increase in the β1-integrin/Tie2 ratio induces a switch to Ang2 signalling via β1-integrin. To investigate Ang2 signalling in the absence of Tie2, we used the HeLa adenocarcinoma cell line, which expresses β1 integrins, but essentially no Tie2 on the cell surface (Supplementary Fig. 6a, endogenous Tie2 staining in HeLa cells marked with an asterisk in Supplementary Fig. 7). Both ectopic Ang2 expression (Supplementary Fig. 6b) and stimulation with recombinant Ang2 (Fig. 4a; Supplementary Fig. 6c) resulted in Ang2 deposition to specific cell–matrix adhesions, very close to active β1-integrin, and this localization was significantly inhibited by β1-integrin-blocking antibodies, but not by treatment with cilengitide^40^, a pentapeptide inhibitor of αv-integrins (Fig. 4a,b; Supplementary Fig. 6c). In contrast, β1-integrin-blocking antibodies did not significantly change the diffuse localization of recombinant Ang1 in Ang1-stimulated cells (Supplementary Fig. 6d). However, when Tie2 was ectopically expressed in HeLa cells, Ang1 was detected in Tie2-positive cell–cell junctions, in line with previous results^13^,^41^, whereas Ang2 localization to cell adhesions occurred both in the presence and absence of ectopic Tie2 or Tie1 (Supplementary Fig. 7). These results indicate that Ang2 deposition in the HeLa-cell matrix is β1-integrin-dependent, but Tie2 independent. Of note, in Ang2-stimulated cells, active β1-integrin was localized to the basal surface, whereas in unstimulated or Ang1-stimulated cells, β1-integrin was found in focal adhesions in the cell periphery (Fig. 4a, quantified in Fig. 4c; Supplementary Fig. 6d).

We next examined whether Ang2 is able to directly activate β1-integrin. We used a sensitive cellular assay measuring the binding of a recombinant fibronectin fragment (FN7–10) to activated endogenous α5β1 integrin in Chinese hamster ovary (CHO) cells relative to total surface β1-integrin expression^42^,^43^. In this assay, Ang2 enhanced CHO-cell binding to fibronectin in a dose-dependent manner, indicative of increased β1-integrin activation (Fig. 4d,e). Of note, a 150 nM (10 μg ml^−1^) concentration of Ang2 stimulated FN7–10 binding to CHO cells equally well as the ectopic expression of the talin head domain, a prominent inside-out activator of β1-integrin^44^. In contrast, Ang1 did not induce β1-integrin activation, even when used in higher concentrations (Fig. 4d,e). Ang2 also directly bound to the β1-integrin subunit in an enzyme-linked immunosorbent assay (ELISA) (Fig. 4f), suggesting that Ang2 activates β1-integrin via a direct interaction.

To investigate the mechanism of Ang2-mediated β1-integrin activation, we created a chimeric Ang2–Ang1 growth factor containing the N-terminal Ang2 superclustering and coiled-coil domains fused to the Ang1 C-terminal fibrinogen-like domain (FLD) (Ang2–Ang1), as well as the opposite Ang1–Ang2 chimera with the Ang1 N-terminal domain attached to the Ang2 FLD (Fig. 4g). The Ang2–Ang1 chimera was localized in cell–matrix adhesions, similarly to full-length Ang2, whereas the Ang1–Ang2 protein showed more diffuse localization when expressed in HeLa cells (Supplementary Fig. 6b). In addition, Ang2–Ang1 activated β1-integrin to the same extent as Ang2, whereas the Ang1–Ang2 chimera had no effect (Fig. 4h). Furthermore, recombinant Ang1 and Ang2 FLDs (residues 245–497 and 242–496, respectively) fused to the Fc region of human immunoglobulin G failed to activate β1-integrin (concentration range 10–50 μg ml^−1^, Supplementary Fig. 6e), whereas expression of the N-terminal domain of Ang2, but not of Ang1, increased the formation of stress fibres in BECs (Supplementary Fig. 6f). These results suggested that the Ang2 N-terminal domain promotes β1-integrin activation, leading to Ang2 deposition in cell–matrix adhesions in a β1-integrin-dependent manner.

**Figure 5:**
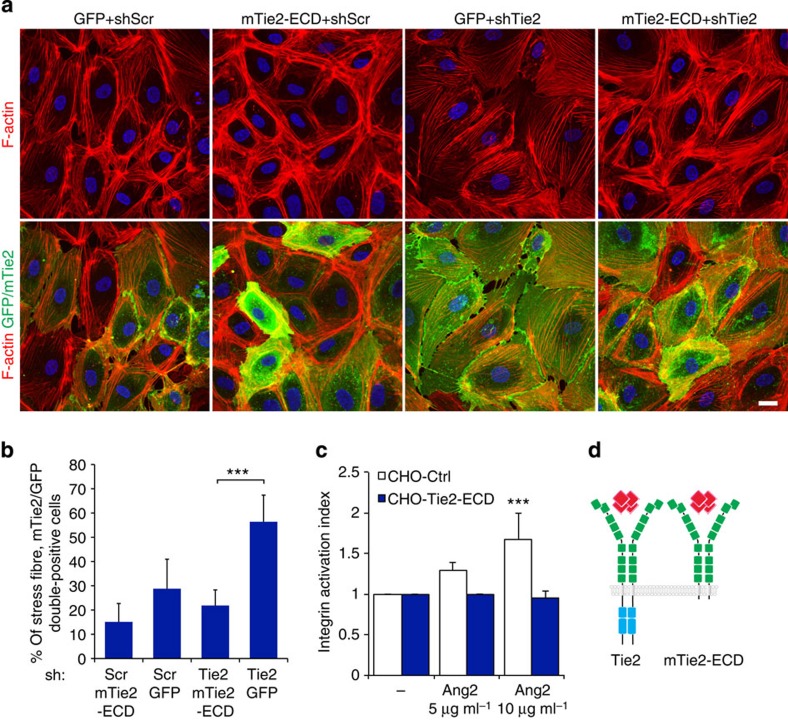
Ang2-mediated β1-integrin activation is inhibited by Tie2 in a kinase-independent manner. (**a**) BECs were transduced with Scr or Tie2 shRNA lentiviruses, with or without vectors expressing the membrane-bound form of either mouse Tie2 ectodomain (mTie2-ECD), or of GFP as a control. The cells were fixed and stained for F-actin and mouse Tie2, or for F-actin only (GFP-transduced samples). (**b**) Quantification of the percentage of stress fibre positive, GFP- or mTie2-positive cells (7 microscopic fields, 400 cells/transduction analysed, *P*=0.002, *n*=3 independent experiments). (**c**) CHO cells transduced with a control vector (CHO-Ctrl) or CHO cells expressing the membrane-bound Tie2 ectodomain (CHO-Tie2-ECD) were incubated with fluorescently labelled fibronectin fragment (FN7–10) and the indicated amounts of rhAng2. FN7–10 binding to CHO and CHO-Tie2-ECD cells, detected by staining for the Tie2 ectodomain, was quantified using fluorescence-activated cell sorting (*P*=0.002 for CHO-Ctrl stimulated with rhAng2 (10 μg ml^−1^) and *P*=0.99 for rhAng2 (10 μg ml^−1^)-stimulated CHO-Tie2-ECD, both compared with FN only control, *n*=3). (**d**) Schematic structures of full-length Tie2 and mTie2-ECD; Ang2 ligand in red. Mean and s.d., Dunnet’s test, ***P<0.005. Confocal microscopic images. 4′,6-Diamidino-2-phenylindole staining of nuclei. Scale bar, 20 μm.

To investigate Tie2 function in Ang2-β1-integrin signalling, we used murine Tie2 receptor complementation in Tie2-silenced BECs. For this, we transduced Tie2-silenced BECs with a retroviral construct expressing the membrane-bound ectodomain of murine Tie2 (mTie2-ECD), which lacks kinase activity (Fig. 5). mTie2-ECD increased the cortical actin cytoskeleton in Tie2-silenced cells, whereas a membrane-bound GFP control protein did not (Fig. 5a,b). Furthermore, Ang2-mediated integrin activation was completely blocked by mTie2-ECD expression, as measured by the fibronectin fragment-binding assay (Fig. 5c). These results indicated that Tie2 captures Ang2 at the endothelial cell surface to inhibit Ang2 signalling via β1-integrin. Furthermore, Tie2 kinase activity was not required for stabilization of the cortical actin cytoskeleton in endothelial cells.

### Changes in the aortic endothelium of Ang2 transgenic mice

In our previous studies, induced expression of murine Ang2 in the endothelium of double-transgenic mice (VE-cadherin-tTA (VEC-tTA)/Tet-OS-Ang2 mice; VEC-tTA/Ang2 for short) resulted in increased lung metastasis, and marked capillary changes in vessels adjacent to extravasated tumour cells, including endothelial cell-basement membrane detachment, reduced endothelial cell–cell junctions and gaps between the endothelial cells^19^. These changes were much less prominent in wild-type (WT) tumour-bearing littermates^19^, suggesting that increased Ang2 levels aggravated endothelial destablization induced by the tumour cells in the lungs.

**Figure 6:**
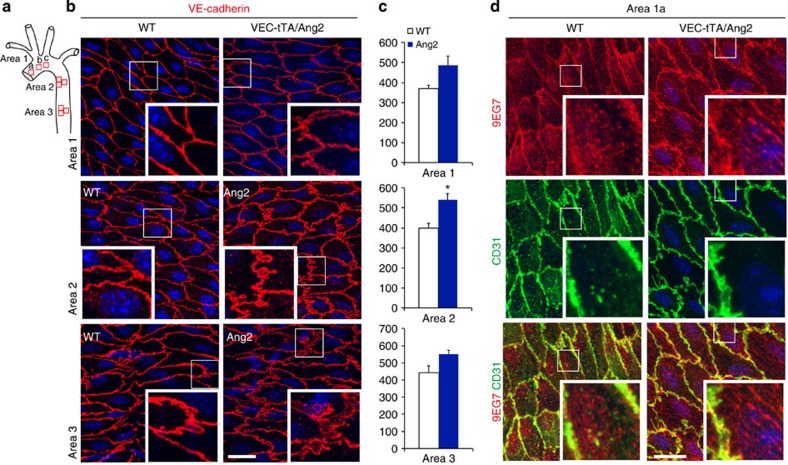
Irregular endothelial cell–cell junctions and increased β1-integrin activation in the aortic endothelium of VEC-tTA/Ang2 mice. (**a**) Schematic illustration of the mouse aorta and the different areas (1–3) analysed. (**b**) Representative *en face* stainings of VE-cadherin in VEC-tTA/Ang2 transgenic or WT littermate mouse aortic endothelium from the areas indicated. (**c**) Quantification of VE-cadherin. Note the trend of increased VE-cadherin area in the VEC-tTA/Ang2 (Ang2) transgenic mice (statistically significant in area 2. *P*=0.03, 3 microscopic images/area, *n*=3 mice/genotype). (**d**) Representative images of active β1-integrin stained with mab 9EG7 in the aortas of VEC-tTA/Ang2 and WT mice. Note increased central localization of elongated active β1-integrin-positive matrix adhesions in the VEC-tTA/Ang2 aortas when compared with WT aortas (*n*=4 mice/genotype). Mean and s.d., Student’s *T*-test, *P<0.05. Projections of confocal z-stacks. Scale bars, 20 μm.

**Figure 7:**
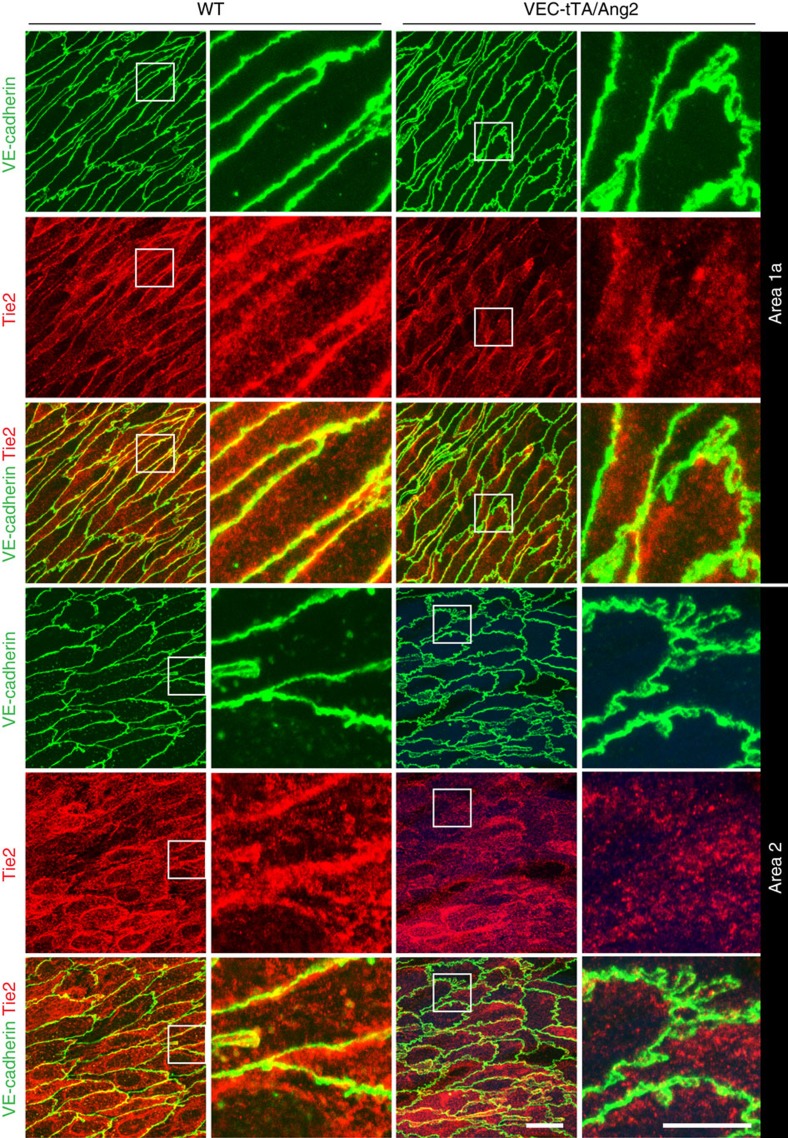
Localization of Tie2 in the aortic endothelium of wild-type and VEC-tTA/Ang2 mice. Representative *en face* stainings of VE-cadherin and Tie2 in VEC-tTA/Ang2 transgenic or WT littermate mouse aortic endothelium from the areas indicated, *n*= 3 VEC-tTA/Ang2 and *n*=4 WT mice. Projections of confocal z-stacks. Scale bar, 20 μm, in magnification 10 μm.

We therefore investigated possible vascular changes that may predispose to compromised barrier function in transgenic mice, in which Ang2 expression was induced after birth. Transgenic Ang2 expression by endothelial cells resulted in elevated systemic Ang2 levels in this model (Supplementary Fig. 8a). To investigate the effects of Ang2 on endothelial integrity in quiescent vessels, we analysed *en face* prepared mouse aorta using whole-mount staining for VE-cadherin, CD31, active β1-integrin, filamentous actin and Tie2 (Figs 6 and 7; Supplementary Fig. 8b). The overall VE-cadherin and CD31 patterns in the cell–cell contacts of WT mouse aorta vary from narrow linear lining in the high-flow regions in the ascending aorta (area 1a) and the outer curvature of the aortic arch^45^, to a more irregular VE-cadherin staining in the descending part (area2 and 3), which is subject to lower-flow forces (Fig. 6a–c). In all regions analysed, the VEC-tTA/Ang2 mice showed a more irregular VE-cadherin staining, when compared to WT or single-transgenic littermates with interdigitating structures emerging at cell–cell junctions. These finger-like structures were also stained by the CD31 antibodies (Fig. 6d). Interestingly, in the VEC-tTA/Ang2 mice, active β1-integrin was localized in central elongated adhesions in the aortic endothelial cells unlike in WT mice, where active β1-integrin-positive adhesions were weakly detected in the cell centre (Fig. 6d). Furthermore, cortical actin staining co-localized with VE-cadherin staining in the aortic endothelium of WT mice, whereas in the VEC-tTA/Ang2 mice, central actin fibres were detected, but they did not overlap with VE-cadherin (see Supplementary Fig. 8b). Notably, Tie2 was enriched in the cell-cell junctions, especially in the high-flow regions of the ascending aorta (area 1a) and in the outer curvature of the arch, but this was reduced in VEC-tTA/Ang2 mice (Fig. 7). These results indicate that elevated Ang2 levels reduce junctional Tie2 localization and alter β1-integrin activation and F-actin and VE-cadherin localization in the otherwise quiescent mouse aortic endothelium, recapitulating *in vivo* the effects of increased Ang2-β1-integrin signalling observed in Tie2-silenced cultured endothelial cells.

Here, we identify Ang2 as an activator of β1-integrin in endothelial and non-endothelial cells, and in the vessel endothelium *in vivo*. Our results show that the autocrine, endothelial cell-secreted Ang2 has profound signalling functions, which do not require Tie2 kinase activity or the context-dependent Tie2 antagonist activity of Ang2. The membrane-bound ectodomain of Tie2 was sufficient to completely block Ang2-induced β1-integrin activation, and to compensate for the silencing of endothelial Tie2 in BECs, suggesting that Tie2, by acting as a ligand trap, inhibited endothelial Ang2-β1-integrin signalling.

The N-terminal super-clustering and coiled-coil domains of Ang2 promoted β1-integrin activation, whereas the C-terminal angiopoietin-FLD-Fc fusion proteins did not, implying a unique Ang2 N-terminal-dependent role in the regulation of β1-integrin function. Ang2 bound directly to the β1-integrin subunit, suggesting a direct activation mechanism of β1-integrin heterodimers. However, our results do not rule out additional interactions with β1-integrin, mediated via the C-terminal domains of Ang2 or Ang1.

Integrin α5β1 and αvβ3 have been previously reported to converge on signalling pathways mediated by Ang1-Tie2 and Ang2-Tie2 complexes, respectively, resulting in different cellular outcomes in endothelial cells^33^,^34^. Our results differentiating between Ang1- and Ang2-mediated β1-integrin activation in the absence of Tie2 are in line with Ang2-integrin signalling in the endothelial tip cells, which express high Ang2 but low Tie2 levels^27^. Thus, besides the differential regulation of Tie2 phosphorylation^6^,^13^,^46^,^47^,^48^, integrin activation is a major dichotomous difference between the signalling mechanisms of Ang1 and Ang2. This fundamental finding should explain some of the perplexing and context-dependent functions of Ang2.

Our results demonstrated that β1-integrin has a dual, subcellular localization-dependent function in the maintenance of the endothelial actin cytoskeleton: β1-integrin in the cell periphery maintained the cortical actin cytoskeleton, whereas centrally located β1-integrin-containing elongated adhesions were associated with actin stress fibres in the Tie2-silenced endothelial cells. Importantly, the localization of active β1-integrin in the elongated adhesions was dependent on Ang2. A recent report demonstrated that activation of β1-integrin due to loss of its inhibitor, ICAP-1, in human cerebral cavernous malformations, results in β1-integrin deposition in fibrillar adhesions, increased actin stress fibres and reduced endothelial barrier function^35^, suggesting that increased β1-integrin activity destabilizes intercellular junctions via increased cell contractility and aberrant pericellular matrix remodelling, in line with our results. Integrins αvβ3 and αvβ5 have been reported to reciprocally regulate the endothelial actin cytoskeleton and contribute to endothelial and pulmonary vascular barrier function in a positive and a negative manner, respectively^37^,^39^. We found that αv- or β3-integrin silencing resulted in increased stress fibre formation and loss of endothelial cortical actin cytoskeleton, in line with previous results^39^. However, αvβ3 or αvβ5-integrins were not required for stress fibre formation in the Tie2-silenced cells, although the reduced junctional localization of αvβ3-integrin and increased stress fibre localization of αvβ5 may further promote the destabilized phenotype of these cells.

Our results from the VEC-tTA/Ang2 transgenic mice indicate that high Ang2 levels regulate endothelial β1-integrin-containing cell-basement membrane adhesion sites, the actin cell cytoskeleton and endothelial cell–cell junctions. Although the mechanism remains to be fully elucidated, our results show that Tie2, especially in the cell junctions, is reduced in the VEC-tTA/Ang2 mice compared with WT mice, which may sensitize the endothelium towards Ang2-β1-integrin signalling. Collectively, these changes should lead to endothelial cell–cell junctions that are predisposed to undergo further destabilization under stress when the endothelium is exposed to, for example, inflammatory cytokines or extravasating tumour cells^12^,^14^,^19^,^32^. This is in line with previous results of the function of Tie2 during endothelial dysfunction, showing that heterozygous Tie2 mice are more susceptible to endotoxin-induced lung injury, and that Tie2 levels are downregulated during septic shock^10^,^49^. Furthermore, reduced Tie2 expression was recently associated with susceptibility to vascular complications induced by haemorrhagic Ebola virus infection in mice^50^. We found that antibodies blocking the Ang2–Tie2 interaction reduced tumour cell transendothelial migration, in line with previously published *in vivo* data^19^. On the other hand, autocrine Ang2-β1-integrin pathway activation in Tie2-silenced BECs resulted in increased transmigration of tumour cells. High Ang2 levels and decreased Tie2 levels may augment Ang2-β1-integrin signalling, endothelial β1-integrin activation and cellular tension, eventually resulting in reduced barrier function. In summary, our results establish Ang2 as an activator of β1-integrin and call for a better understanding of the Ang2-β1-integrin pathway, when blocking reagents targeting Ang2 are developed for the treatment of human diseases, including cancer.

## Methods

### Reagents and cell culture

Human dermal microvascular blood endothelial cells (BECs, PromoCell, Heidelberg, Germany, or Lonza, Basel, Switzerland) were maintained in endothelial basal medium (ECBM, PromoCell or EBM-2) with fetal bovine serum (FBS) and growth supplements, provided by the manufacturers, on 1 μg ml^−1^ fibronectin-coated culture plates. CHO, HeLa and LLC cells (ATCC) were maintained in Dulbecco’s modified Eagle’s medium (DMEM) (Lonza), and NCI-H460-N15 ATCC (LNM-35 for short) in RPMI (Lonza), all media supplemented with 2 mM L-glutamine, penicillin (100 U ml^−1^), streptomycin (100 μg ml^−1^) and 10% FBS. LNM-35 and LLC cells were made fluorescent (LNM-35-GFP) by the expression of the GFP^19^. Packaging cell lines 293-GPG VSV-G^51^ (growth medium: DMEM glucose 4.5 g l^−1^ supplemented with 10% FBS, 1% glutamine, 0.2% penicillin, 0.2% streptomycin, 0.2% puromycin, 0.6% neomycin and 1 μg ml^−1^ tetracycline) and 293FT (growth and transduction medium: DMEM glucose 4.5 g l^−1^ supplemented with 10% FBS, 1% L-glutamine, 0.2% penicillin and 0.2% streptomycin) were transduced for retrovirus (transduction medium: DMEM glucose 4.5 g l^−1^, 20 mM HEPES, supplemented with 10% FBS, 1% L-glutamine, 0.2% penicillin and 0.2% streptomycin) and lentivirus production with Fugene 6 (Roche, Basel, Switzerland), respectively. Retroviral constructs were cloned into the pMXs vector (generous gift from Dr Kitamura, University of Tokyo, Japan). For angiopoietin stimulations, the HeLa cells were starved for 2 h in 2% FBS–DMEM, and stimulated in the starvation medium using 60 nM (4 μg ml^−1^) rhAng1 and rhAng2 (R&D Systems, Minneapolis, MN) for 30 min. mAb13 (BD Biosciences, San Jose, CA) was used at 4 μg ml^−1^.

The following antibodies were used at dilution 1:100 for immunofluorescence (IF) staining, unless otherwise indicated: anti-hTie1 (AF619), anti-hTie2 (AF313, WB 1:4,000), anti-m/r-Tie2 (AF762, 1:1,000), anti-hAng2 (AF623) (R&D Systems), anti-mTie2/Tek4 (cat. 95–585, 1:80, Millipore, Billerica, MA), anti-hVE-cadherin (1:200, cat. 555661, Pharmingen, BD Biosciences; cat. 2500, Cell Signaling Technology, Danvers, MA), anti-mVE-cadherin (555289; BD Biosciences or 14–1441, eBioscience, San Diego, CA), anti-His-tag (1:40, 2365, Cell Signaling Technology), anti-Flag (1:1,000, F3648, M2, Sigma-Aldrich, St Louis, MO), anti-fibronectin (F3648, Sigma-Aldrich), anti-β1-integrin (1:30)^52^, mab12G10 (1:300, Abcam, Cambridge, UK), mab2252 (Merck Millipore) and anti-hamster α5β1 (1:50, PB1, developed by Brown and Juliano^53^ and obtained from the Developmental Studies Hybridoma Bank developed under the auspices of the NICHD and maintained by the University of Iowa), anti-mouse CD31 (1:2,000, Mab1398Z, Millipore), anti-αvβ3 (1:40, mab1976, Millipore), Alexa-488, Alexa-594, Alexa-647-conjugated secondary antibodies (1:300, Life Technologies, Carlsbad, CA) and anti-hamster FITC (Jackson Immunoresearch, Westgrove, PA). Filamentous actin was stained using Texas red or Alexa-488-conjugated phalloidin (1:200, Invitrogen, Life Technologies). For immunoblotting, cells were lysed in M-PER lysis buffer (Pierce)^54^. Uncropped immunoblots and larger blot areas are presented in the Supplementary Fig. 9.

### Q-RT–PCR

Total RNA was isolated using the RNA NucleoSpin II Kit (Macherey-Nagel), reverse transcribed to complementary DNA (cDNA) using the SuperScript VILO cDNA Synthesis Kit (Invitrogen) or the RT^2^ First Strand Kit (Qiagen) and used (10 ng RNA eqvivalent/reaction) for Q-RT–PCR. Primers used were: Ang1 (5′-aacatgggcaatgtgcctacactt-3′ and 5′-cattctgctgtatctgggccatct-3′ or 5′-acgtggaaccggatttctct-3′ and 5′-tttagtacctgggtctcaacatct-3′), Ang2 (5′-cagattttggaccagaccagtga-3′ and 5′-tcaatgatggaattttgcttgga-3′), Ang4 (5′-atccagcgccgtgagaatg-3′ and 5′-aaatgttcgtactgggcatagg-3′ or 5′-caggactgtgcagagatcca-3′ and 5′-tctccgaagccctgtttgta-3′). An estimate of Ang2 messenger RNA copy number was calculated based on control samples of known Ang2 cDNA concentrations. Integrin expression was analysed using the RT^2^ profiler Array Wound Healing (Qiagen).

### shRNA and siRNA silencing of cells

Cells were transduced with shRNA lentivirus or retrovirus particles in the presence of 0.1% Polybrene (Sigma-Aldrich) for 48 h. For small interfering RNA (siRNA) silencing of Tie2, BECs were starved overnight in 1% FBS endothelial medium, without supplements, and transduced twice for 24 h on subsequent days, with siRNA against Tie2 or scramble sequences (SC-36678, Santa Cruz Biotechnologies) using Oligofectamin (Invitrogen). shRNA lentiviruses from the TRC1 library were used together with packaging plasmids pCMVg and pCMVdelta8.9. Unless otherwise indicated, shRNA#4 was used to silence Tie2, and shRNA#27 for Ang2 silencing.

### Immunofluorescence staining

Cells were fixed for 10–15 min in 4% paraformaldehyde (PFA)–PBS, washed 3 × 5 min with PBS, permeabilized 5 min with 0.1–0.2% Triton X-100 in PBS, blocked 10 min in 1% bovine serum albumin (BSA)–PBS, and incubated 30 min with primary antibodies in 1% BSA–PBS, washed with PBS, blocked and subsequently incubated for 30 min with secondary antibodies at room temperature, washed with PBS and mounted using DAPI-Vectashield (Vector laboratories Inc, CA, USA) or Mowiol-Dabco (Sigma-Aldrich); nuclei were prestained with Hoechst (Sigma-Aldrich).

### Quantification of microscopic images

Images were captured using a Zeiss digital Axiocam camera connected to a Zeiss Axioplan 2 microscope and a × 40 oil objective, or using a laser scanning confocal microscope (Zeiss LSM 780 or Zeiss LSM 5 Duo) with a × 63 or a × 40 oil objective. Three-dimensional projections were digitally reconstructed from confocal z-stacks. Stress fibre-positive cells were identified by the presence of stress fibres extending across the longitudinal cell axis, while cells with cortical actin showed no actin staining in the cell centre surrounding the nucleus. Stress fibre-positive cells were imaged using an epifluorescent microscope with a × 40 objective and counted from 3–7 randomly selected epifluorescence micrographs per experiment. In each image, the number of stress fibre-positive cells was divided by the total number of cells based on nuclear staining. Two independent investigators counted the stress fibre-positive cells in an investigator-blinded fashion. Quantification of the VE-cadherin area was performed from similarly acquired epifluorescence micrographs and the Image J software ( http://imagej.nih.gov/ij/).

### Adhesion assay

BECs were transduced with scramble or Tie2 shRNA lentiviruses for 48 h. Cells were detached with trypsin-EDTA and let to recover in EBM-2 complete medium for 30 min. A total of 15,000 cells were seeded on each coverslip, precoated with 10 μg ml^−1^ of fibronectin for 1 h and let to adhere for 45 min. Coverslips were fixed and subjected to immunofluorescence staining. For quantification of increased adhesion maturation, pixel area of active integrin β1-positive adhesion structures was quantified in the central 50% of the total cell area and normalized to total area in the central 50% of the cell (see squares in Fig. 2b).

### Tie2 complementation assay

BECs were grown to 50% confluence and transduced for 24 h with lentiviral shRNA against Tie2 or scramble sequences. The cells were additionally transduced with retroviruses coding for membrane-anchored mouse Tie2 ectodomain (mTie2-ECD) or membrane-anchored GFP for another 24 h. Cells were fixed and mTie2-ECD-transduced samples were stained using specified antibodies against mouse Tie2. mTie2-ECD- or GFP-positive cells with stress fibres were quantified from three independent experiments, seven epifluorescence micrographs/experiment, imaged using × 40 objective.

### Tumour cell–endothelial cell transmigration assay

Tumour cell transmigration across an endothelial cell monolayer was performed using Transwell chambers (6.5 mm insert diameter, 8 μm pore size, Corning Life Sciences, NY, USA). BECs (100,000), transduced with Tie2 or scramble shRNA lentiviruses, were seeded on the upper compartment overnight, after which 100,000 LLC-GFP were applied on top of the confluent endothelial monolayer for 9 h, with complete growth media in both chambers. Inserts were fixed in 4% PFA–PBS and mounted onto glass slides. Five microscopic fields per insert were imaged at × 40 magnification using the Zeiss Axioplan microscope, and transmigrated cells were quantified from the bottom of the filter as the GFP-positive area using the ImageJ software.

Alternatively, 100,000 LNM-35-GFP cells were applied on top of the confluent BEC monolayer for 5 or 9 h and treated with control or anti-Ang2 antibody blocking the Ang2–Tie2 interaction. The inserts were fixed and imaged using the Zeiss AxioVert200 connected to Zeiss AxioCam with × 10 magnification from the centre of the bottom of the insert. GFP-positive LNM-35 cells were counted manually from three independent experiments, each performed as triplicate.

### Integrin activation assay

For testing chimeric angiopoietin proteins, CHO cells were left untreated or transfected at 25–30% confluency with 5,000 ng of plasmid constructs for Ang1–Ang2–Flag, Ang2–Flag or Ang2–Ang1–Flag with lipofectamine 2000 (Life Technologies) preincubated with Opti-MEM (according to the manufacturer’s instructions). After 24 h, the transduction mixture was changed to serum-free alpha-MEM (Minimum Essential Media) (2 mM L-glutamine). The next day, part of the culture medium was collected, the cells were detached with HiQtase in serum-free alpha-MEM, spun and resuspended to respective culture mediums collected earlier. Each sample was subsequently treated with 1% Alexa-647-conjugated fibronectin fragment (repeats 7–10, FN7–10) and 10% anti-hamster α5β1 antibody (PB1), recognizing total α5β1 irrespectively of α5β1 activation state. For testing of recombinant proteins, CHO cells were collected as mentioned with HiQtase in serum-free alpha-MEM, and preincubated 30 min with various concentrations of rhAng2, rhAng1 or EDTA, treated with 1% Alexa-647-conjugated fibronectin fragment, incubated for 30 min at room temperature, washed with PBS, fixed with 4% PFA for 15 min, washed and stained with the total α5β1 antibody. All samples were then stained with Alexa-488-conjugated anti-mouse secondary antibody to detect the anti-α5β1 antibody and analysed using fluorescence-activated cell sorting. The results were expressed as the activation index of β1-integrin: the ratio between the activated and total β1-integrin. This was calculated by measuring integrin activity (with Alexa-647-labelled fibronectin fragment binding) against a background signal (labelled FN-binding in the presence of inactivating EDTA) normalized against total cellular β1-integrin.

### Fibronectin matrix remodelling

BECs were plated on vitronectin (Invitrogen)-coated coverslips and transduced with scramble or shTie2 lentiviruses. Cells were maintained in endothelial growth medium, supplemented with serum, depleted for fibronectin by rotating the serum with gelatine sepharose (GE Healthcare, Fairfield, CT, USA) two times for 1 h, and spinning the sepharose containing serum using Micro Bio-Spin chromatography columns (Bio-Rad, Hercules, CA, USA). Depletion of fibronectin was confirmed using western blotting.

### Elisa assay for β1-integrin binding to Ang2

Maxisorp plate was coated with anti-Flag antibody (13 μg ml^−1^) in Hepes-buffered saline (HBS) containing 1 mM CaCl2, 20 μl per well and incubated at room temperature for 4 h. The wells were washed twice with HBS, followed by incubation with 10 μl of concentrated conditioned media from control or Ang2-expressing 293 T cells in 50 μl HBS overnight at room temperature. The wells were washed twice, blocked with 2% BSA for 2 h at room temperature, washed twice and incubated with 1:5 dilutions of biotinylated β1-integrin ectodomain (ECD), 20 μl per well, for 4 h at room temperature. The wells were washed twice, incubated overnight at +4 °C with Streptavidin-HRP (Dako) in 2% BSA, 1:4,000 dilution, washed 3 × and followed by incubation with the horseradish peroxidase substrate. Absorbance was measured using the Multiscan Ascent spectrophotometer (Thermo Labsystems) at 450 nm.

### Expression vector cloning

Angiopoietin chimeras Ang1–Ang2–Flag and Ang2–Ang1–Flag were constructed in the following fashion: the angiopoietin FLD and the adjacent N-terminal linker region (amino acids R262-F498 in Ang1; K249-F496 in Ang2) were changed between Ang1 and Ang2 using two-step PCR and cloned into the pMXS vector. The constructs were tagged with a Flag-peptide-coding sequence (DYKDDDDK), which was attached directly to the chimeric angiopoietin C terminus, followed by a stop codon. Ang2 and Ang2–Ang1 contained the native Ang2 signal sequences, whereas Ang1 and Ang1–Ang2 were expressed under the Igκ light-chain signal sequence. The N-terminal Ang1 (amino acids 1–261) and Ang2 (amino acids 1–248) forms with C-terminal Flag-tag were cloned by PCR into the pMXs vector. mTie2-ECD was cloned by PCR into the pMXs vector, resulting in a deletion of the intracellular I824-A1124 amino acids in mTie2. A membrane-bound form of enhanced GFP was created by attaching a myristoylation and palmitoylation sequence from the Lyn kinase (MGCIKSKRKDNLNDDGVD)^55^ to the enhanced GFP N terminus by PCR. The resulting PCR fragment was inserted in pMXs.

### Animal models

Mice were maintained in the Laboratory Animal Centre of the University of Helsinki. The National Animal Experiment Board in Finland approved animal experiments used in this study. We used the VEC-tTA/Tet-OS-Ang2 mouse line, which expresses mouse Ang2 under an inducible endothelial cell promoter^19^. The driver VEC-tTA and responder transgenic mouse lines were bred together to obtain double-transgenic VEC-tTA/Tet-OS-Ang2 offspring. To overcome the embryonic lethality due to endothelial Ang2 overexpression in double-transgenic embryos, Ang2 expression was repressed during the entire pregnancy. Tetracycline (Sigma-Aldrich) at 2 mg ml^−1^ in 5% sucrose was added to the drinking water of pregnant females, starting at the time of mating and until birth, when Ang2 expression was induced by discontinuation of tetracycline administration. Single-transgenic or WT littermates were used as controls for double-transgenic mice, both designated as WT. None of the control mice displayed any obvious phenotype.

### *En face* preparation and whole-mount staining of mouse aorta

For *en face* preparation of aortas, 2–8-month-old mice were anaesthetized with intraperitoneal injections of xylazine (10 mg kg−1) and ketamine (80 mg kg−1) and perfusion fixed with 1% PFA–PBS. Aortas were fixed for 1 h in 1% PFA–PBS, washed with PBS and blocked with donkey immunomix (5% donkey serum, 0.2% BSA, 0.3% TritonX-100 and 0.05% sodium azide in Dulbecco’s PBS) for 1 h at room temperature. Whole-mount staining was performed using indicated primary antibodies for 48 h+4, followed by extensive washing using 0.3%-TritonX-100–PBS at room temperature, incubation with secondary antibodies for 16 h, washing and post-fixing of the samples. The aortas were mounted in DAPI-Vectashield and z-stacks were obtained using Zeiss LSM 780 and a × 63 oil objective. Maximal projections of VE-cadherin-positive stacks were used for VE-cadherin quantification.

### Statistical tests

Student’s *t*-test (two tailed, unequal variance) was used for pairwise comparisons, and Dunnet’s test for multiple comparison analysis. A *P* value <0.05 was considered statistically significant.

## Author contributions

L.H. performed experiments, analysed the data, prepared figures, participated in experimental design and in writing of the manuscript. T.S. and V.-M.L. designed and performed some of the experiments. P.G. performed some of the experiments with endothelial cells. H.N. helped with the *in vivo* experiments. L.E. performed transmission electron microscopy. J.I. and G.J. participated in experiments with β1-integrin. The Ang2 transgenic mouse studies were done in collaboration with K.A., and K.A. and J.I. participated in writing the manuscript. P.S. designed the experiments, supervised L.H., T.S. and P.G., analysed the data, prepared the figures and wrote the manuscript.

## Additional information

**How to cite this article:** Hakanpaa, L. *et al.* Endothelial destabilization by angiopoietin-2 via integrin β1 activation. *Nat. Commun.* 6:5962 doi: 10.1038/ncomms6962 (2015).

## Supplementary Material

Supplementary Information Supplementary Figures 1-9, and Supplementary Methods.
