# Supplementary material for: Endothelial destabilization by angiopoietin-2 via integrin β1 activation
Source: Nat Commun. 2015 Jan 30;6:5962. doi: 10.1038/ncomms6962 (PMC4316742; doi:10.1038/ncomms6962)
Supplement: Supplementary Information — Supplementary Figures 1-9, and Supplementary Methods. [file ncomms6962-s1.pdf]

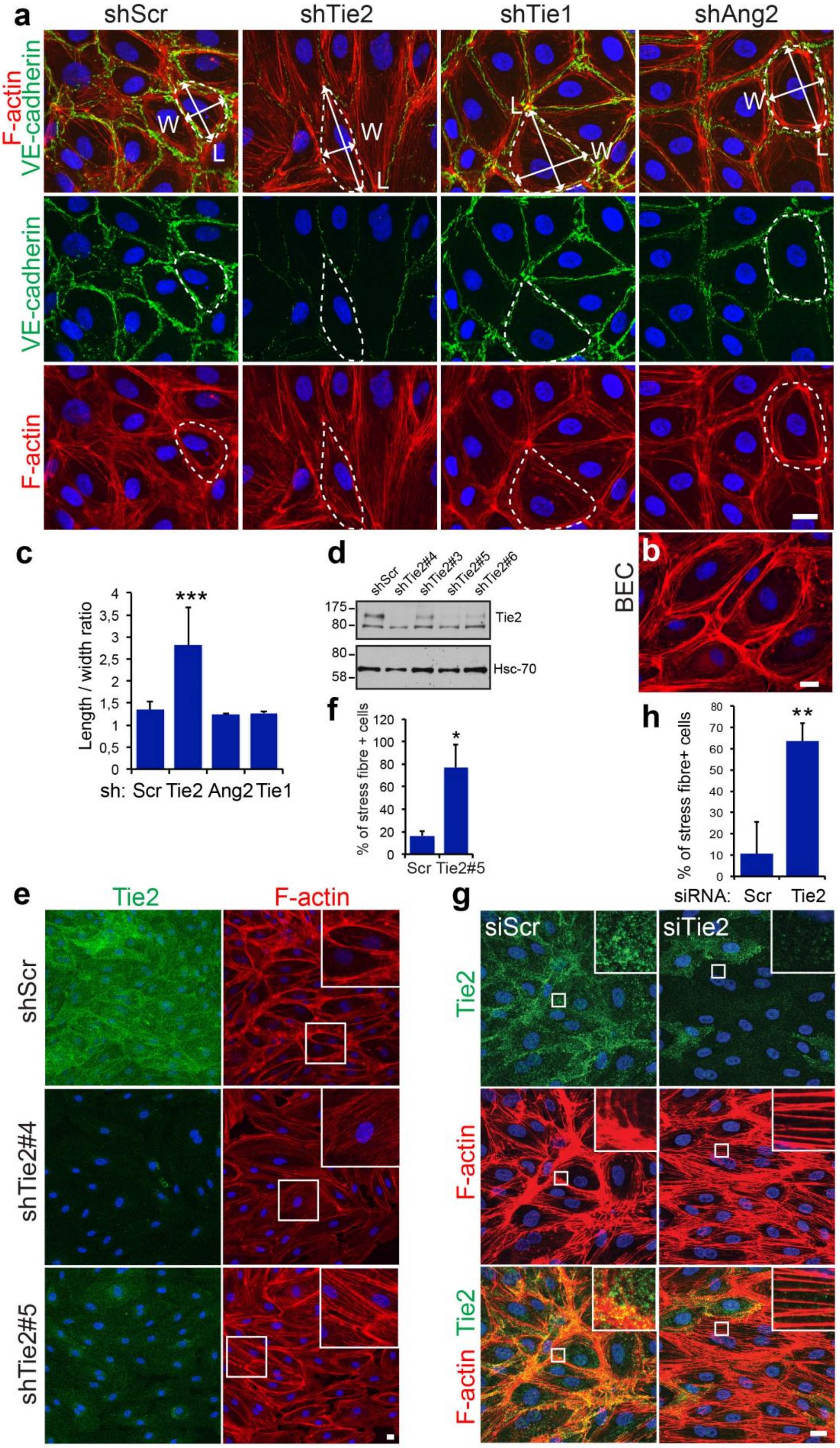

a
shScr
shTie2
shTie1
shAng2
F-actin VE-cadherin
VE-cadherin
F-actin
W
L
b
BEC
c
Length / width ratio
***
sh: Scr Tie2 Ang2 Tie1
d
shScr
shTie2#4
shTie2#3
shTie2#5
shTie2#6
175
80
Tie2
80
58
Hsc-70
f
% of stress fibre + cells
*
Scr Tie2#5
h
% of stress fibre+ cells
**
siRNA: Scr Tie2
e
Tie2
F-actin
shScr
shTie2#4
shTie2#5
g
siScr
siTie2
Tie2
F-actin
Tie2 F-actin

Supplementary Figure 1. Silencing of Tie2, but not of Tie1 or Ang2, induces stress fibre formation and elongation of microvascular endothelial cells. **a.** BECs were transduced with scramble (Scr), Tie2, Tie1 or Ang2 shRNA lentiviruses, fixed and stained for F-actin and VE-cadherin. **b**. Untreated BECs grown for 48 hours were fixed and stained for F-actin. **c.** The cell length to cell width ratio was quantified, representatively shown in (a) (total of 150 cells/shRNA analysed, n=3 independent experiments, *P*=0.002, Dunnet's test). W=cell width, L=cell length **d.** BECs silenced with 4 different Tie2 shRNA lentiviruses were analysed in Western immunoblotting for Tie2, and Hsc-70. **e.** BECs were silenced with Scr or two different Tie2 shRNA lentiviruses (#4 and #5 from c), and stained for Tie2 and F-actin. **f**. Quantification of the percentage of stress fibre positive cells in shScr and shTie2#5 lentivirus transduced BECs (total number of cells analysed: 300 cells/Scr and 183/Tie2#5 shRNA, n=3 independent experiments, *P*=0.03, Student's T-test). **g-h.** BECs were transfected with Scr or Tie2 siRNA, and stained for Tie2 and F-actin (g). The percentage of stress fibre positive cells was quantified from a representative experiment, repeated 3 times (total number of cells 180 cells/siRNA analysed, *P*=0.007, Student's T-test) (h). Mean and standard deviation. * $P<0.05$, ** $P<0.01$, *** $P<0.005$. Confocal microscopic images. DAPI staining of nuclei. Scale bars: 20 μm.

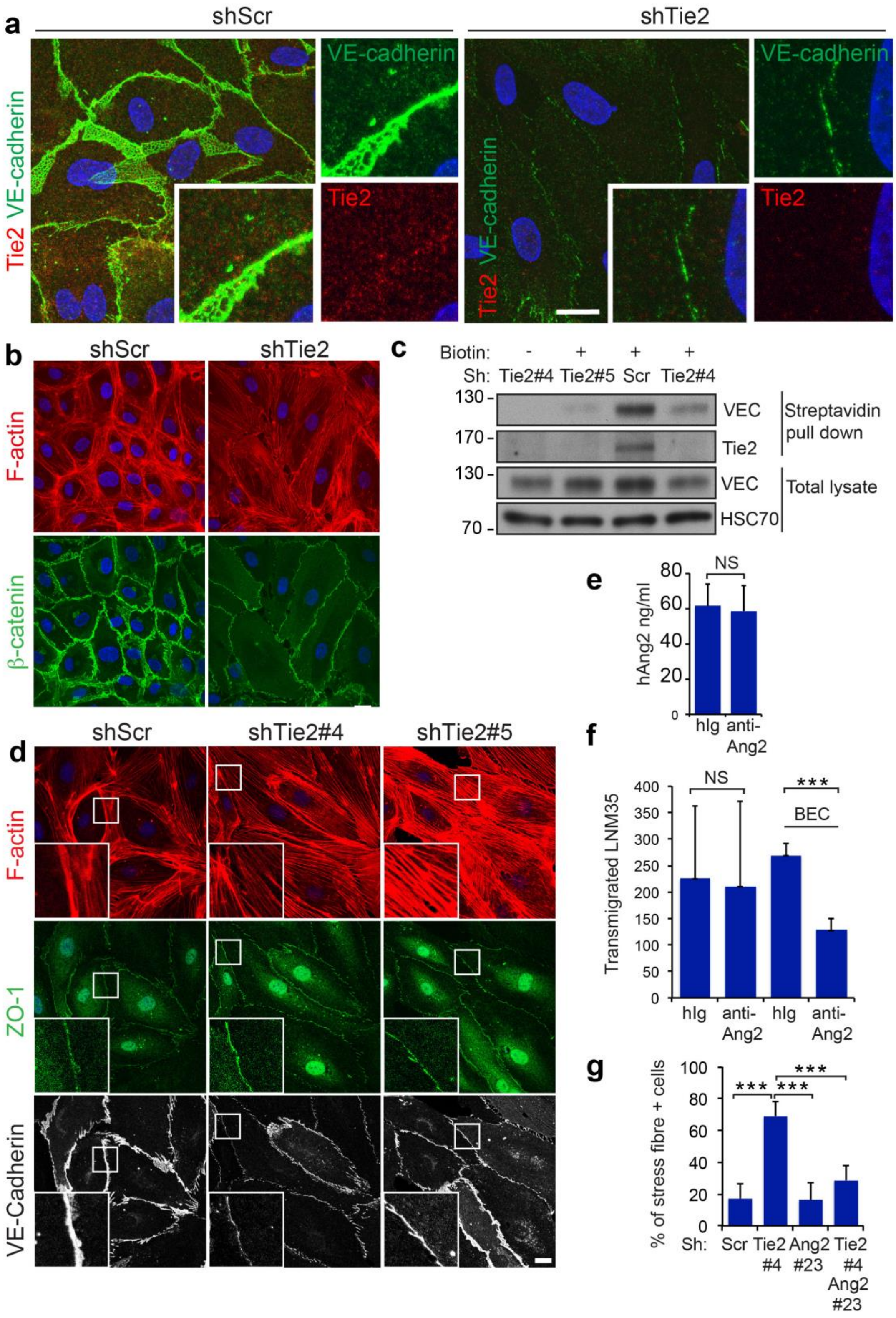

Supplementary Figure 2. Altered adherens junctions in Tie2-silenced endothelial cells. **a.** Human pulmonary microvascular endothelial cells were transfected with scramble (Scr) or Tie2 shRNA lentiviruses, fixed and stained for Tie2 and VE-cadherin. **b.** BECs were transfected with Scr or Tie2 shRNA lentiviruses, fixed and

stained for Tie2 and β-catenin. **c.** BECs silenced with scr or 2 different Tie2 shRNA lentiviruses were cell-surface labelled with biotin, and the streptavidin pull-downs were analysed in Western immunoblotting for VE-cadherin and Tie2. Total cell lysates were analysed for VE-cadherin and Hsc-70. **d.** BECs were silenced with Scr or two different Tie2 shRNA lentiviruses, and stained for F-actin, ZO-1 and VE-cadherin. **e.** Conditioned media of BECs grown on the Transwell filters was analysed for hAng2 using ELISA. **f**. LNM-35 migration was analysed across the Transwell inserts in the absence and presence of a BEC monolayer, but in both cases in the BEC conditioned medium. P =0.9 in the absence and P =0.001 in the presence of a BEC monolayer, n=3, Student's T-test. **g**. BECs were transduced with Scr, Tie2, Ang2#23 or Tie2+Ang2#23 shRNA lentiviruses, fixed, and stained for F-actin. Quantification of the percentage of cells displaying actin stress fibres (number of cells analysed/ lentivirus shRNA transduction: 605/shScr; 464/shTie2; 555/shAng2#23; 527/shTie2+shAng2#23. P=0.0013 for shTie2 vs. shScr; P=0.004 for shTie2 vs. shAng2; P=0.0041 for shTie2 vs. shTie2+shAng2, n=3 independent experiments, Dunnet's test). Mean and standard deviation, *** P<0.005, NS=not significant. Confocal microscopic images, (a) and (d) are projections of confocal z-stacks. DAPI staining of nuclei. Scale bars: 20 μm.

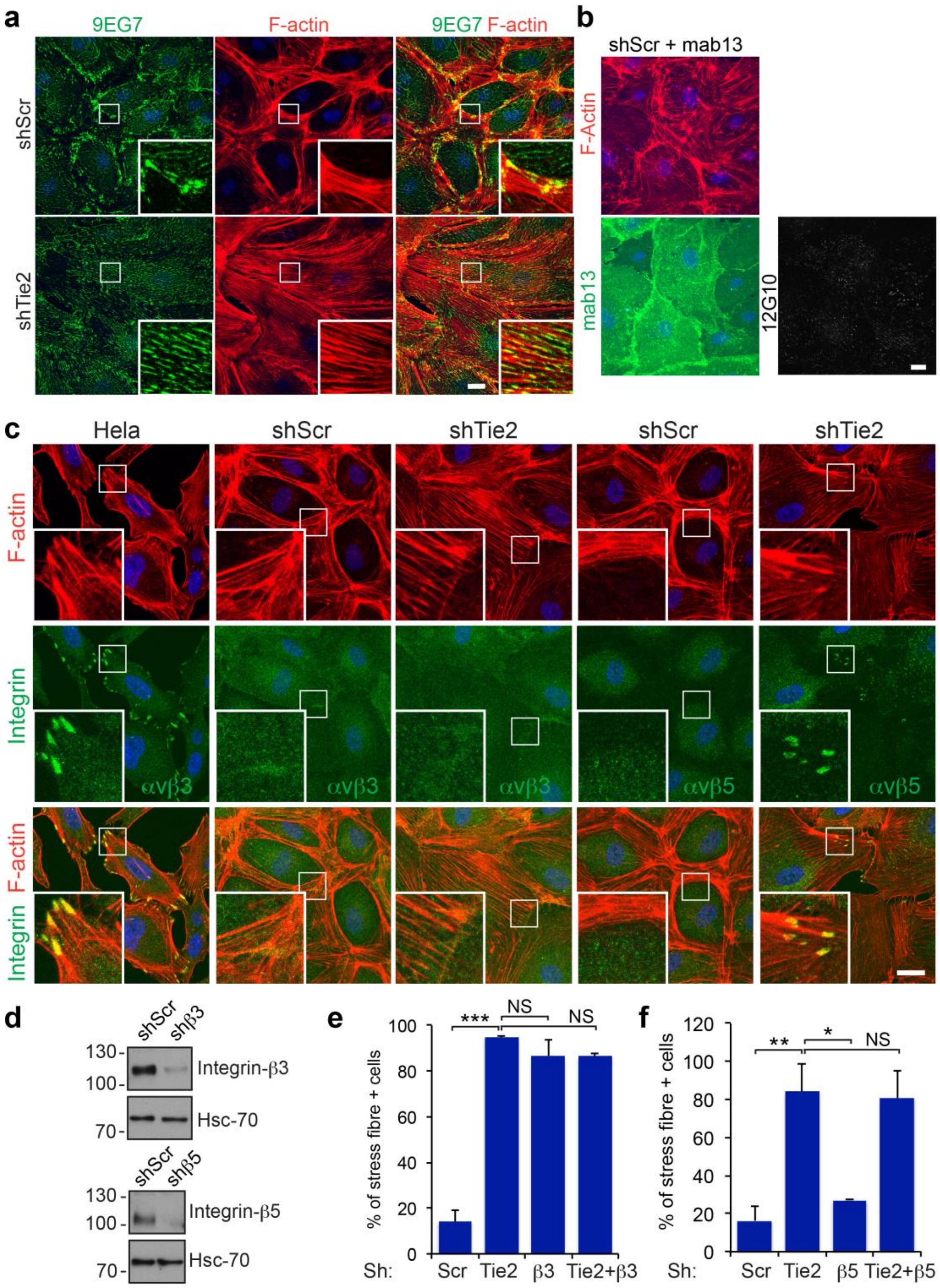

Supplementary Figure 3. Localisation and effect of β1, αvβ3 and αvβ5-integrin inhibition in control and Tie2-silenced BECs. **a.** BECs were transduced with Scr or Tie2 shRNA lentiviruses, fixed, and stained for F-actin and active integrin β1 (9EG7). **b.** BECs were transduced with Scr shRNA lentiviruses for 48 hours, and treated with β1-integrin blocking antibodies (mab13) (10 μgml^{-1}) during 32-48 h after transduction and stained for F-actin, mab13 and active β1-integrin (12G10). Tie2 shRNA silenced

cells are shown in Fig. 3c. **c.** BECs transduced with Scr or Tie2 shRNA lentiviruses, or HeLa cells as a control, were fixed, and stained for F-actin and αvβ3- and αvβ5-integrins. **d**. BECs transduced with Scr, β3-integrin or β5-integrin shRNA lentiviruses were analysed in Western blot, as indicated. **e-f**. BECs were transduced with Scr, Tie2, β3-integrin (e) or β5-integrin (f) or with Tie2 + β3- or β5-integrin shRNA lentiviruses, fixed, and stained for F-actin, and the percentage of cells displaying actin stress fibres was quantified. **e**. Number of cells analysed/lentivirus shRNA transduction: 359/shScr; 154/shTie2; 197/ shβ3-integrin; 192/shTie2+shβ3-integrin. *P*=0.001 for shTie2 vs. shScr; P=0.299 for shTie2 vs. shβ3-integrin; P=0.277 for shTie2 vs. shTie2+shβ3-integrin. **f**. Number of cells analysed/lentivirus shRNA transduction: 343/shScr; 175/shTie2; 210/shAng2#23; 136/shTie2+shβ5-integrin. *P*=0.008 for shTie2 vs. shScr; P=0.015 for shTie2 vs. shβ5-integrin; P=0.9775 for shTie2 vs. shTie2+shβ5-integrin. Mean and standard deviation, * *P*<0.05, ** *P*<0.01, *** *P*<0.005, n=2 independent experiments, Dunnet's test. Hoechst (a) and DAPI (c) staining of nuclei. Confocal microscopic images, (a) and (c) are projections of confocal z-stacks. Scale bars: 20 μm.

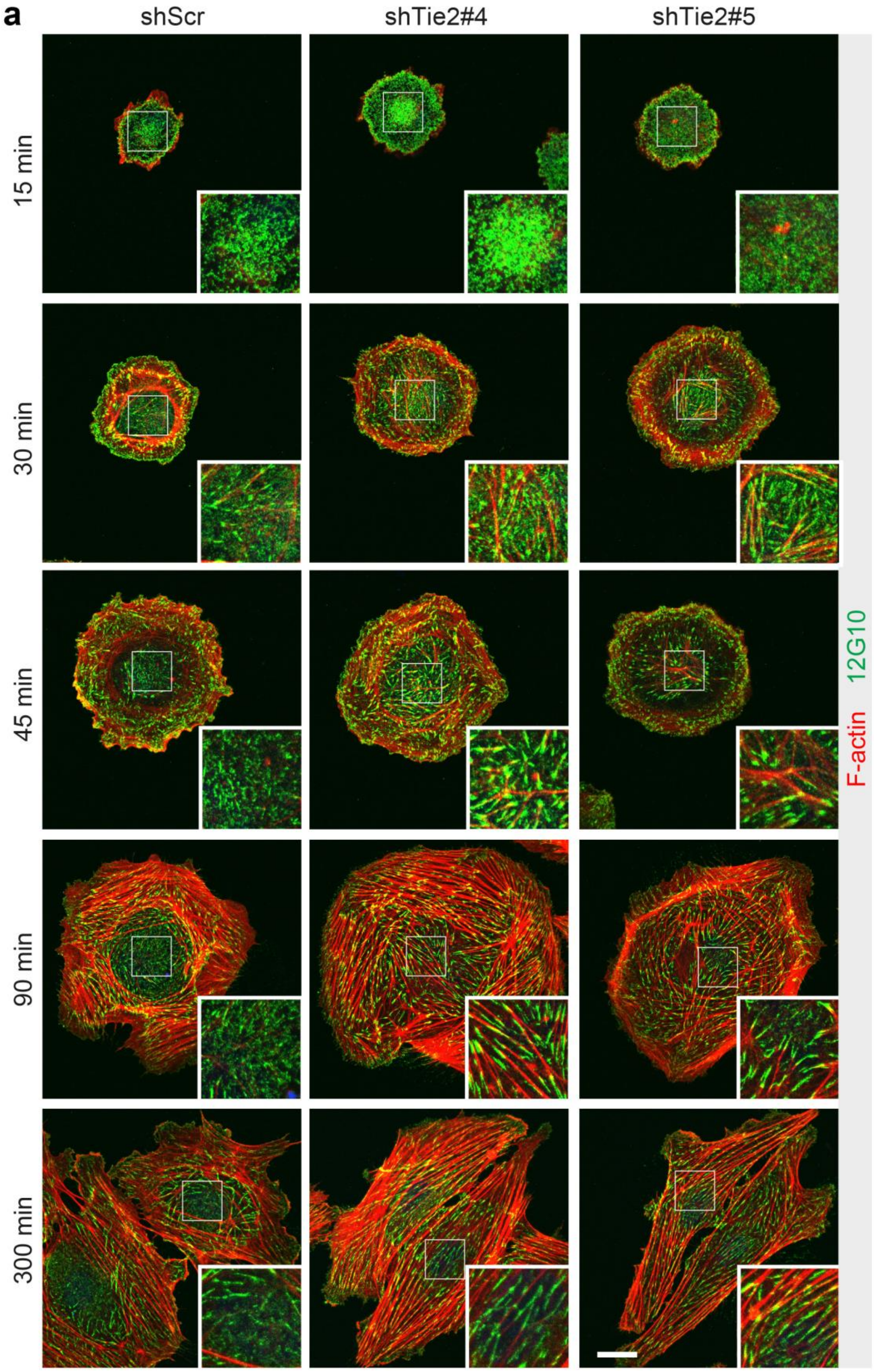

a
shScr
shTie2#4
shTie2#5
15 min
30 min
45 min
90 min
300 min
F-actin
12G10

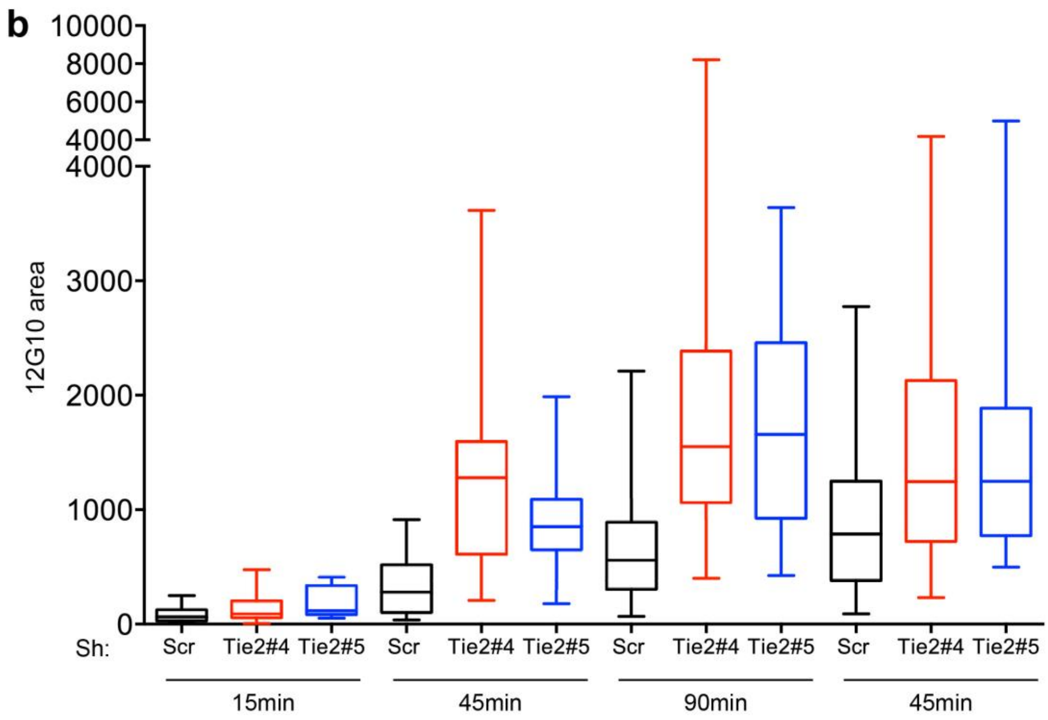

Supplementary Figure 4. Time course of spreading of Scr or Tie2 shRNA lentivirus transduced BECs on fibronectin. **a**. Fixed cells were stained for F-actin and active β1-integrin (12G10). **b**. β1-integrin positive matrix adhesion sites in the central 50% of total cell area were quantified from a representative experiment in a. (10 40x microscopic images and 30 cells/time point/shRNA analysed). Results from 3 independent experiments at a 30 min time point are shown in Figure 2c. Mean and standard deviation. Confocal microscopic images. Scale bar: 20 μm.

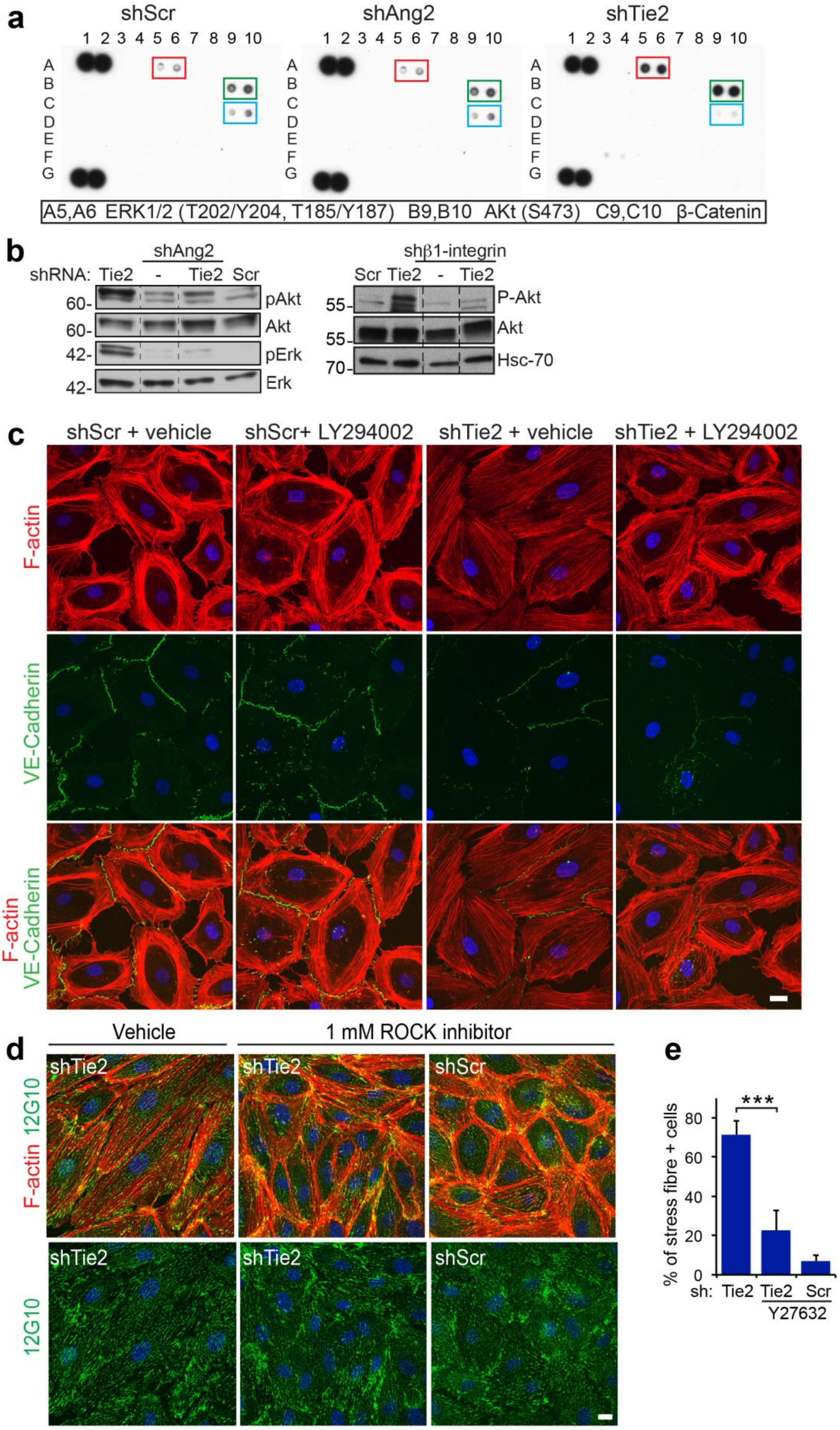

a
shScr
shAng2
shTie2
1 2 3 4 5 6 7 8 9 10
A B C D E F G
A5,A6 ERK1/2 (T202/Y204, T185/Y187) B9,B10 AKt (S473) C9,C10 β-Catenin
b
shRNA: Tie2 shAng2 - Tie2 Scr
pAkt
Akt
pErk
Erk
60-
60-
42-
42-
shβ1-integrin
Scr Tie2 - Tie2
P-Akt
Akt
Hsc-70
55-
55-
70-
c
shScr + vehicle
shScr+ LY294002
shTie2 + vehicle
shTie2 + LY294002
F-actin
VE-Cadherin
F-actin VE-Cadherin
d
Vehicle
1 mM ROCK inhibitor
shTie2
shTie2
shScr
F-actin 12G10
12G10
e
% of stress fibre + cells
0 20 40 60 80
***
sh: Tie2 Tie2 Scr
Y27632

Supplementary Figure 5. Activation of the PI3K-Akt and Rho kinase pathways in Tie2-silenced endothelial cells. **a.** BECs were transduced with the indicated shRNA lentiviruses, lysed and analysed using phosphoproteomic blots. Phosphorylation of Erk and Akt were increased in Tie2-silenced cells, when compared to either scramble or Ang2-silenced cells. **b.** BECs were transduced with the indicated shRNA lentiviruses and the cell lysates were analysed by immunoblotting for phospho-Akt (S473), total Akt, phospho-Erk (T202, Y204), total Erk and HSC-70. **c.** BECs were transfected with Scr or Tie2 shRNA lentiviruses, and after 48 hours treated with the PI3 kinase (PI3-K) inhibitor LY294002, or vehicle for 30 min. The cells were fixed and stained for F-actin and VE-cadherin. **d-e.** BECs were transfected with Scr or Tie2 shRNA lentiviruses, and after 48 hours treated with the Rho kinase (ROCK) inhibitor (Y27632), or vehicle for 2 hours. The cells were fixed and stained for F-actin and active integrin β1 (12G10) (d). The percentage of stress fibre positive cells was quantified from a representative experiment in (e), repeated 3 times (total of 150 cells/treatment, P=0.004, Student's T-test). The mean and standard deviation. *** P<0.005. Hoechst staining of nuclei. Confocal microscopic images. Scale bars: 20 μm.

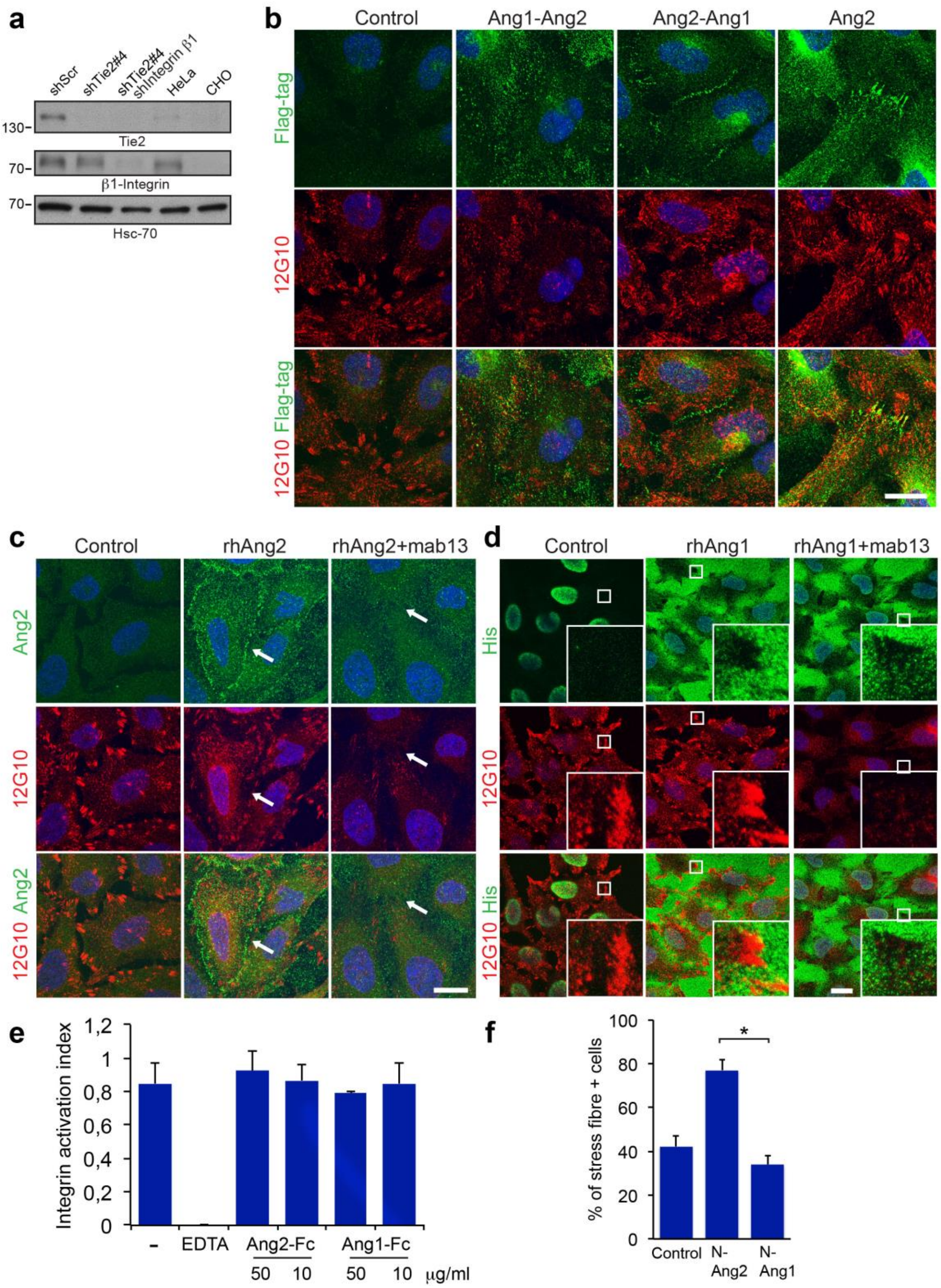

Supplementary Figure 6. Deposition of Ang1 and Ang2 in HeLa cell matrices, and the effect of C- and N-terminal angiopoietin domains on β1-integrin activation and stress fibres. **a.** Western blot of HeLa, CHO and shRNA lentivirus silenced BEC lysates for Tie2, β1-integrin and HSC-70. **b.** HeLa cells were transduced with retroviral vectors coding for Ang2-Flag or chimeric Ang1-Ang2-Flag or Ang2-Ang1-Flag proteins, fixed and stained for active β1-integrin (12G10) and for the

angiopoietin proteins using anti-Flag antibodies. **c-d.** HeLa cells were treated with β1-integrin blocking antibody mab13 for 5 minutes or left untreated, and then further stimulated for 30 min with rhAng2 (c) or rhAng1 (d) (both 4 μg/ml), fixed and stained using antibodies against Ang2 (c) or His-tag (Ang1, d) and active β1-integrin (12G10). **e**. CHO cells were incubated with fluorescently labelled fibronectin fragment (FN7-10) and with EDTA, as a control, or with various concentrations of Ang2-FLD-Fc or Ang1-FLD-Fc fusion proteins containing the fibrinogen-like domains (FLD) of Ang2 and Ang1, respectively, fused to the Fc domain of IgG, as indicated. FN7-10 binding to CHO cells was quantified using fluorescence activated cell sorting, and normalized to total α5β1 levels, as explained in the materials and methods. (n=2). **f**. BECs were transduced with retroviral vectors coding for N-terminal domains of Ang2 (N-Ang2; amino acids 1-248) and Ang1 (N-Ang1; amino acids 1-261), and the percentage of stress fibre positive cells was quantified (cells/transduction: 260/control; 224/N-Ang2; 230/N-Ang1, n=2, P=0.01, Student's T-test). The mean and standard deviation, * $P<0.05$. Nuclear Hoechst/DAPI stain. Projections of confocal z-stacks. Scale bars: 20 μm.

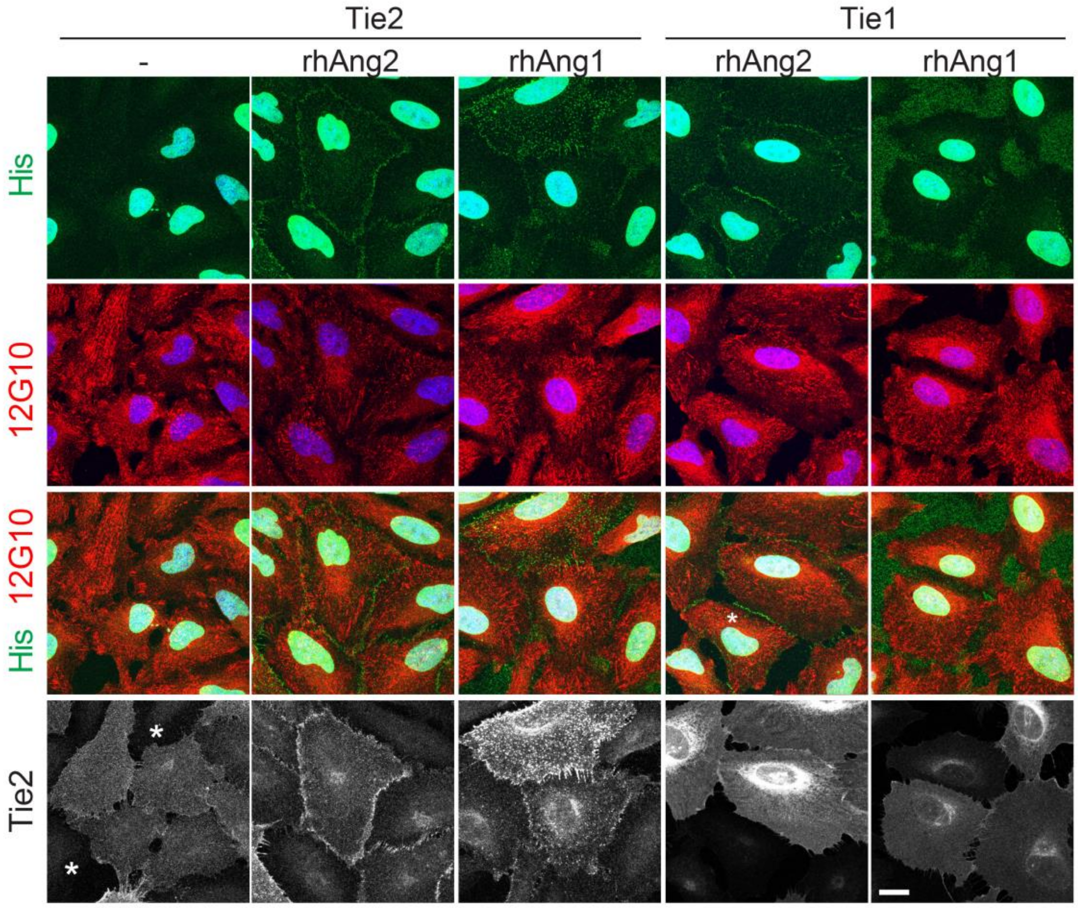

Supplementary Figure 7. HeLa cells were transduced with retroviral vectors coding for Tie2 or Tie1, as a control, and stimulated with rhAng2 or rhAng1, fixed and stained for active β1-integrin (12G10), His-tag and Tie1 or Tie2. Asterisks indicate non-transduced cells, where Tie2 immunoreactivity is concentrated around the nuclei. Nuclear DAPI stain. Projections of confocal z-stacks. Scale bar: 20 μm.

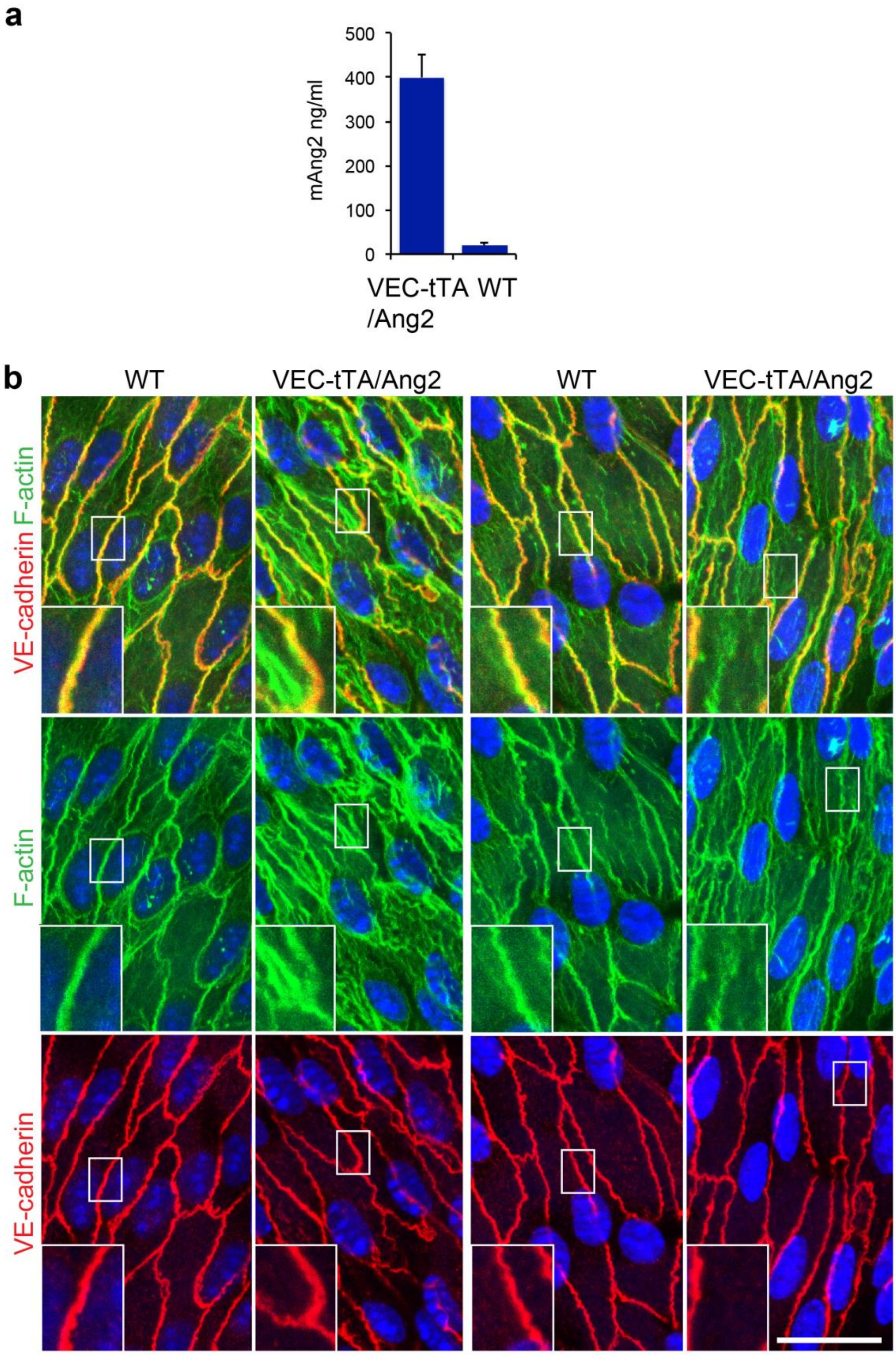

Supplementary Figure 8. Ang2 serum levels and aortic endothelial actin staining in WT and VEC-tTA/Ang2 transgenic mice. **a.** Ang2 was quantified by ELISA from the serum samples of VEC-tTA/Ang2 double transgenic and WT littermate control mice.

Mean and standard deviation (n=5/DTG, n=10/WT). **b.** *En face* analysis of the aortic endothelium of WT and VEC-tTA/Ang2 double transgenic mice for filamentous actin and VE-cadherin. Projections of confocal z-stacks. Scale bar: 20 μm.

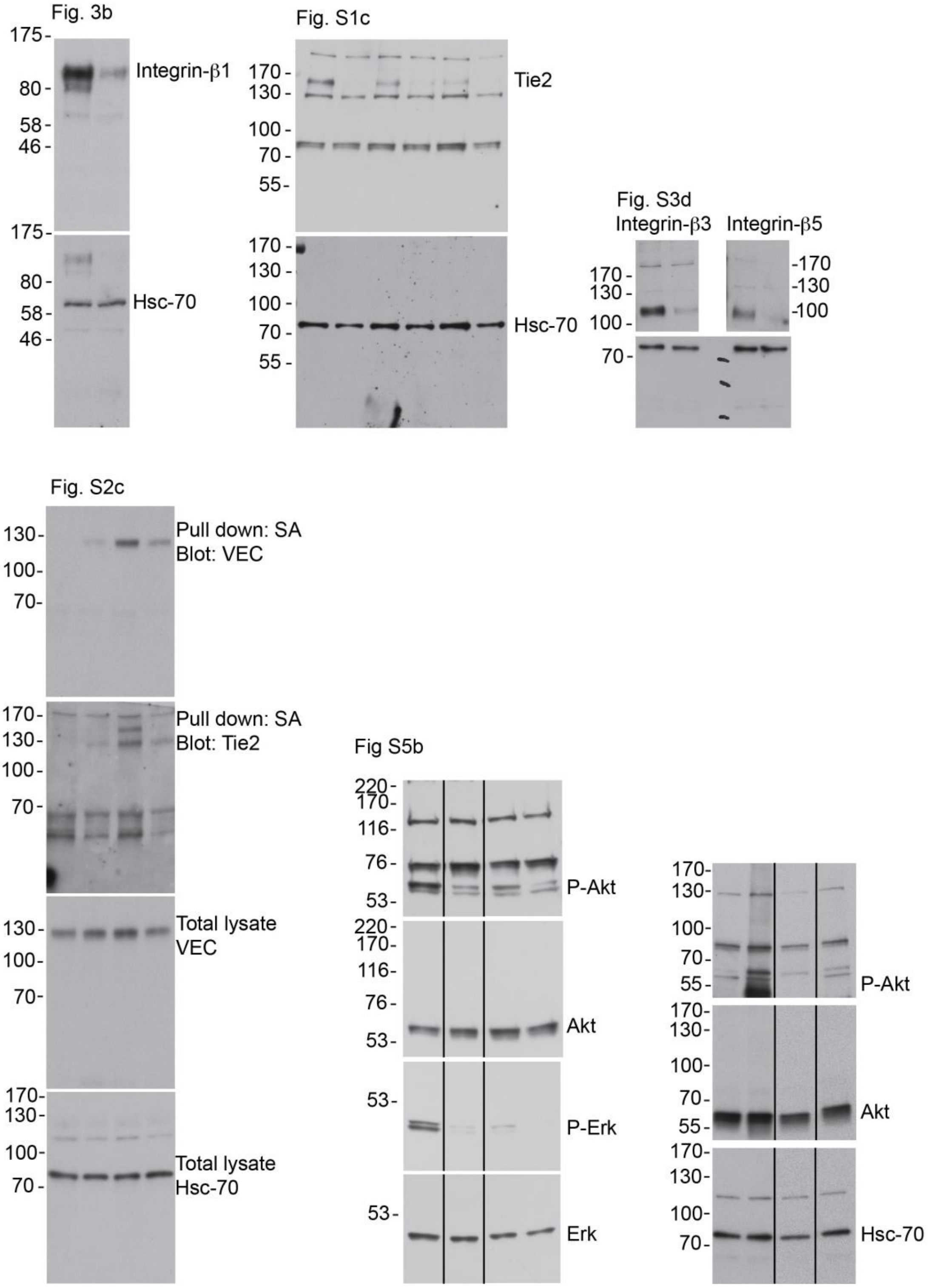

Supplementary Figure 9. Uncropped Western blots of the most important blots.

Supplementary methods

Cell culture. Human pulmonary microvascular endothelial cells (HPMECs, PromoCell) were maintained in endothelial basal medium (ECBM, PromoCell), with fetal bovine serum (FBS) and growth supplements, provided by the manufacturers, on 1μg/ml fibronectin coated culture plates.

Image analysis. Quantification of cell length/width ratio was analysed by measuring the longitudinal cell axis from epifluorescent microscopic .tiff images in Photoshop, and dividing it with the shorter cellular axis.

Reagents. Human phospho-kinase array kit (R&D systems) was used to analyse downstream signalling in scramble, Tie2 or Ang2-silenced BECs. Mouse/rat Angiopoietin-2 Quantikine ELISA kit and Human Angiopoietin-2 Quantikine ELISA Kit (R&D systems) were used to determine Ang2 levels in the sera of Ang2 transgenic and WT mice, and in the conditioned media of cultured human cells, respectively. The following antibodies were used: anti-αvβ5 (Millipore), anti-integrin-β3 (ab75872 1:1000, Abcam), integrin-β5 (ab75872 1:500, Abcam), anti-ZO-1 (Invitrogen), mab9EG7 (BD Biosciences), anti-β-catenin (BD Biosciences), P-Akt (9271, 1:1000, Cell Signaling or AF887, 1:1000, R&D Systems), Akt (9272, 1.1000, Cell Signaling), P-Erk (9106, 1:1000, Cell Signaling), Erk (4372, 1:1000, Cell Signaling). PI3-kinase inhibitor LY294002 (Sigma Aldrich) and the Rho kinase inhibitor Y27632 (Abcam) were used as 1 or 10 □M for 30min or 2 hours, respectively, in complete endothelial medium.

Biotinylation of cell surface proteins. shRNA lentivirus transduced BECs were treated with sulfo-NHS-SS-biotin (0.2mg/ml) in Dulbecco's PBS (DPBS) for 30 min at +4°C. The reaction was stopped using 20 mM sodium-2-mercaptoethanesulpnonate (MesNa) in 50mM Tris-HCl pH 8.6, 100mM NaCl for 15min at +4°C, and subsequently changed to 20 mM iodoacetamide in DPBS for 10min at +4°C. After washing with DPBS, the cells were lysed (25 mM Tris pH 7.4, 100 mM NaCl, 2mM $MgCl_2$, 0,5 mM EGTA, 5% glycerol and 1% Triton-X100 supplemented with the protease inhibitor cocktail). The lysates were incubated with Streptavidin-coupled Dynabeads (Life Technologies) for 60 min at +4°C, washed 6x with PBS and boiled in 1xLaemmli sample buffer, and separated in SDS-PAGE. Surface-expressed, biotinylated VE-cadherin and Tie2 were analysed using Western blot.

Protein Expression and Purification

Human angiopoietin-1 and angiopoietin-2 (residues 245-497 and 242-496, respectively) receptor-binding domains were cloned into the pFastBac1 (Invitrogen) baculovirus expression vector with a mellitin signal peptide and a C-terminal Fc-tag. The extracellular domain (ECD) of human integrin β1 (residues 21-728) was cloned to the pFastBac1 baculovirus expression vector with a mellitin signal peptide and a C-terminal hexahistidine-tag. Recombinant baculovirus was produced in Spodoptera frugiperda (Sf9) insect cells grown at +26 ^{o}C in serum-free Insect-Express (Lonza) medium supplemented with 50 μg/ml gentamycin (Sigma). Recombinant baculovirus was harvested 72h post-transfection by centrifugation for 5 min at 4000 x g.

For protein expression, Trichoplusia ni (Tn5) insect cells were grown in about 250 ml of serum-free Insect-Express (Lonza) medium per 1 L shaker flask at +26 ^{o}C and 110 rpm shaker speed. The cell cultures were infected with the corresponding recombinant baculovirus at high multiplicity and at three days post infection, the supernatant was harvested by centrifugation for 20 min at 8000 x g.

The supernatant of the angiopoietin constructs was loaded onto Protein A sepharose (4CLB, GE Healthcare) column with a peristaltic pump. The resin was extensively washed with PBS and the Fc fusion proteins were eluted with 0.1 M Glycine pH 3.0. The supernatant containing the integrin β1 ECD was supplemented with 10 mM Imidazole and 1 mM $NiCl_2$ and was loaded onto Ni^{2+}-charged resin (Ni-NTA Superflow; Qiagen) column with a peristaltic pump. The resin was extensively washed with 20 mM HEPES (pH 7.5) containing 20 mM imidazole and 0.6 M NaCl. The Integrin β1 ECD was eluted with the washing buffer supplemented with 0.4 M imidazole. All of the eluates were further purified by gel filtration on a Superdex 200 (GE Healthcare) column in PBS and concentrated to desired concentration by centrifugal filter units (Amicon Ultra-15 MWCO 10 kDa, Millipore).
